# Make science evolve into a One Health approach to improve health and security: a white paper

**DOI:** 10.1186/s42522-019-0009-7

**Published:** 2020-04-17

**Authors:** Albert D. M. E. Osterhaus, Chris Vanlangendonck, Maurizio Barbeschi, Christianne J. M. Bruschke, Renee Christensen, Peter Daszak, Frouke de Groot, Peter Doherty, Patrick Drury, Sabri Gmacz, Keith Hamilton, John Hart, Rebecca Katz, Christophe Longuet, Jesse McLeay, Gaetano Morelli, Joergen Schlundt, Trevor Smith, Sameera Suri, Khristeen Umali, Jan van Aken, Jaap A. Wagenaar

**Affiliations:** 1 Research Center of Emerging Infections and Zoonoses, University of Veterinary Medicine, Hannover, Germany; 2 One Health Platform, Berlare, Belgium; 3 WHO, Health Emergencies Programme, Geneva, Switzerland; 4 Ministry of Agriculture, Nature and Food Quality, The Hague, The Netherlands; 5 The Global Outbreak Alert and Response Network, World Health Organization, Geneva, Switzerland; 6 Ecohealth Alliance, New York City, USA; 7 Microbiology and Immunology, University of Melbourne, Melbourne, Australia; 8 World Organisation for Animal Health (OIE), Paris, France; 9 Georgetown University Medical Center, Washington D.C., USA; 10 Connecting Organizations for Regional Disease Surveillance (CORDS), San Francisco, USA; 11 Nanyang Technological University, Singapore, Singapore; 12 Global Affairs Canada, Ottawa, Canada; 13 Utrecht University, Utrecht, the Netherlands

## Abstract

The World One Health Congresses are biennial gatherings of approximately 1500 professionals from relevant international organisations, OIE, FAO, WHO, World Bank, leading scientific experts and researchers in the field of One Health, animal production and trade, food safety, animal health, human health and environmentology/ecology, government representatives in public health, human health, food safety, environmental health and global health security. The Congress is organized by the One Health Platform.

This white paper summarizes highlights of the 5th International One Health Congress in Saskatoon, Canada, June 2018 and serves as a roadmap for the future, detailing several concrete action points to be carried out in the run-up to the 6th World One Health Congress in Edinburgh, Scotland, June 2020.

## Executive summary

The World One Health Congresses are biennial gatherings of approximately 1500 professionals from relevant international organisations, OIE, FAO, WHO, World Bank, leading scientific experts and researchers in the field of One Health, animal production and trade, food safety, animal health, human health and environmentology/ecology, government representatives in public health, human health, food safety, environmental health and global health security. The Congress is organized by the One Health Platform.

The meeting of scientists occurs in the **One Health Science (OHS) and Antimicrobial Resistance (AMR) programme tracks**. The programme is based on the Scientific Agendas of OHS and AMR. The aim is to exchange high-level scientific data and to foster collaborations. The exchange between scientists and government officials responsible for public health, animal health, food safety and global health security occurs in the **Science Policy Interface (SPI) programme track**.

This document summarizes some highlights of the 5th International One Health Congress in Saskatoon, Canada, June 2018 and serves as a roadmap for the future, detailing several concrete action points to be carried out in the run-up to the 6th World One Health Congress in Edinburgh, Scotland, June 2020.

In summary, the most important actions coming out of the 5th International One Health Congress are:

- The need for the organization of an International One Health Forum in collaboration with Africa CDC with a focus on 3 questions: 1) What are the top zoonotic diseases of importance across the African continent? 2) How should National Public Health Institutes (NPHI’s) conduct surveillance for a selection of these priority zoonotic diseases? 3) How do NPHI’s assess the risk of zoonotic cases and clusters in collaboration with animal and environmental sectors?
- The need for the establishment of a Bio Threats Scanning Group that comprises top level experts from leading laboratories in pathogen discovery. Their main tasks will be
  - ◦ to provide an overarching perspective to One Health and Global Health Security and as such connect both worlds.
  - ◦ to help predict potential biological events that could impact public health security in the future and to detect gaps in research in the fields of zoonoses, emerging infectious diseases and AMR, including the ecological and environmental factors which impact on these diseases.
- Establishing a focused scientific agenda on OHS and on AMR as a framework of the 6th World One Health Congress, June 2020, Edinburgh and engage the broader scientific community and the public health and global health security communities.
- The need for increased connections between One Health Science and Global Health Security and thus more attention for One Health Science with an impact on Global Health Security in governments and international institutions and subsequently increased inter-sectoral collaboration.
- Further discussion and work on “peace time” preparedness elements: syndrome surveillance in humans and animals, pathogen discovery (identification platforms for humans and animals), diagnostic development and distribution platforms, mathematical modelling, animal models, pathogenesis study platforms for new infections, preventive intervention platforms, therapeutic discovery platforms and communication strategies.
- The need for the establishment of a network of spokespersons on science-related vaccine issues that unites vaccination advocates worldwide

## Introduction: make science evolve to improve health and security

There is a significant increase in the emergence of infectious agents and the risk of new pandemics as exemplified by the spread of highly pathogenic H5N1 influenza since 2003, the pandemic H1N1 influenza in 2009, influenza H7N9 in 2013, SARS, MERS, chikungunya, dengue, Zika and Ebola. It is relevant to note that SARS, as the first novel pandemic threat of the new millennium, has clearly demonstrated that previously unknown pathogens can emerge from a wildlife source at any time in any place and without warning, threaten the health, well-being and economies of all societies. There was a clear need for countries to have the capability and capacity to maintain an effective alert and response system to detect and quickly react to outbreaks of international concern, and to share information about such outbreaks rapidly and transparently. Responding to pandemic threats requires global cooperation and global participation. Combined with the growing globalization of health risks and the importance of the human-animal-ecosystem interface in the evolution and emergence of pathogens, **the best solution appears to be a One Health approach.**

It is very clear that zoonoses are an International Public Health issue: in the past two decades, 60% of emerging infectious human diseases had their source in animals. Since 1970, new infectious diseases of humans have been discovered at an average rate of 1 every 8 months. Influenza pandemics are an economic issue: the World Bank has suggested that a low level pandemic could globally reduce production by almost 1% of gross domestic product, a moderate pandemic by almost 2% and a serious pandemic by as much as 5%, which would result in a serious economic recession. Zoonotic diseases that start to spread among humans are also a societal issue: SARS in 2003 and H1N1 influenza in 2009 have shown how quickly panic, stigmatization and mistrust towards governments and the scientific community can arise (even during clearly moderate epidemics). Effects may be long-lasting and have long-term consequences for populations’ support of health measures. This emphasizes the important role of communication in health issues. Zoonotic diseases have security implications: 80% of known biological weapons have a zoonotic origin. Antimicrobial resistance develops and spreads at the animal human interface and is a major challenge to the future health of mankind. According to the World Bank the cumulative impacts by 2050 are $100 trillion and 10 million human deaths annually. Agriculture and aquaculture contribute to direct transmission of resistant strains and AMR dispersion, reduced efficacy threatens both health and food production.

The One Health Platform was established in 2015 and is a not for profit foundation under Belgian law. It is a One Health Scientific Reference Network that aims to enhance understanding of, and preparedness for current and future outbreaks of zoonoses, emerging and re-emerging infectious diseases in humans and animals and antimicrobial resistance. This includes the ecological and environmental factors which drive and impact on these diseases.

As a Scientific Reference Network, the Platform unites some of the best One Health science researchers and global experts in its Scientific Advisory Board. In order to serve the community, the One Health Platform has built strategic alliances with industry through its Industry Advisory Board and its partners through the One Health Coalition. Ties with governments are being secured by the establishment of a Governments Group. They form an informal think tank to safeguard a true exchange of ideas, needs and information bridging science and policy. The Governments Group consists of government officials responsible for public health, animal health, environmental health, food safety and global health security.

As such the Platform is a Strategic Forum of Stakeholders, with the aim of constructing connections across One Health Science and One Health Policy - safeguarding Global Health Security. The One Health Platform strives to achieve gender equality in all internal committees, congress committees, events and projects.

## Epidemics and outbreaks

### HIGHLIGHT: addressing zoonotic diseases at the animal-human-ecosystem interface - future Earth’s top ten challenges for one health

*Peter Daszak, Ecohealth Alliance*

A One Health approach can help to understand the ecological and epidemiological dynamics that shape disease risks, directly inform disease prevention and control strategies, help examine threats and opportunities, and broaden our capacity to proactively address them.

Future Earth’s One HEALTH Global Research Project conducts an annual horizon scan review of hot topics for One Health that will profoundly change the future of health on the planet. These are topics deemed to have serious potential implications for health, have been under-emphasized or are emerging issues, and can benefit from new thinking informed by a One Health perspective.

#### Big data, artificial intelligence and monitoring earth systems

Over the next 5 years, the way data is collected, manipulated, and interpreted will fundamentally change the way the earth’s natural systems will be monitored [1]. This seismic shift is being led and funded by large organizations, supported by NGOs, governments, and academia. The outcome of this combined effort has been described as an artificial intelligence (AI) platform for the planet, with applications ranging from monitoring land use change to detecting disease pathogens. The possibility to combine data from a wide range of applications and domains changes how problems can be framed and how potential solution pathways can be identified and applied. It can improve monitoring of earth systems with subsequent improvements in decision making and long-term policy development and can improve detection of novel potential pathogens.

#### The global virome project

The Global Virome Project (GVP) represents a general trend in pathogen discovery whereby rapid, cheap sequencing coupled with better understanding of wildlife-host disease ecology is leading to a revolution in viral discovery. The GVP’s goals are to discover over 70% of the currently unknown viral diversity in mammals and water birds that normally harbor viruses that could potentially cause disease in people. This will revolutionize our ability to understand risk of emerging diseases, predict where and when pathogens could emerge, and design new mitigation strategies, including avoidance behaviors, vaccines and drugs. The background ecological and anthropological data collected by the GVP and other projects will contribute to our knowledge of wildlife biodiversity and distribution, with potential positive conservation benefits.

#### Mitigating the underlying drivers of EIDs

Emerging infectious diseases (EIDs) are almost entirely due to anthropogenic drivers such as socioeconomic and environmental changes. Our current strategies to deal with EIDs as a major public health threat rely on vaccines, drugs and behavioral intervention after they emerge. This has led to a series of large-scale outbreaks causing high mortality, morbidity and significant economic shocks (e.g. the West African Ebola virus outbreak) leading to mobilization of significant resources in response. Analysis of global trends in EIDs suggest that they are increasing in a non-linear manner and that their economic impact is increasing exponentially [2]. Thus, new strategies to deal with their underlying drivers would be more cost-effective as a strategy to combat this rise in pandemic risk.

#### Gene editing technology

Gene editing technology has become increasingly accurate and affordable in recent years with the advent of the CRISPR-CAS9 gene editing system. The most immediate benefits center around treating, curing, and preventing genetic and non-communicable disease in humans, but the implications for global health go far beyond editing the human genome. About 750 million people live in extreme poverty in the world. Harnessing gene editing to modify livestock and agricultural products may lead to disease resistant domesticated plants and animals, as well as to more productive varieties that can survive and thrive in unusual and changing climates. However, this technology has potential challenges and opportunities that require a One Health lens to help assess and balance risks and benefits.

#### Anthropogenic evolution and synanthropic species

Humans influence the evolution of species and characteristics through artificial selection and domestication processes. In addition, human activity drives the selection process, and creates environments that favor species tolerant to modern civilization. Antibiotics, translocation of exotic species, wildlife trade, pollution, and deforestation are additional anthropogenic activities that are contributing to biodiversity loss. We are also seeing evidence of vectors, reservoir hosts, and pathogens that were influenced by human activity in the past 10,000 years emerging as more problematic and resilient infectious diseases [3]. These newly configured synanthropic species will emerge as significant health and biodiversity challenges in modern landscapes.

#### Mechanisms and manifestations of disease transmission

Although significant strides have been made in discovering the underlying mechanisms of infectious disease manifestation and transmission, more research is necessary to understand how these mechanisms differ under various ecological and anthropogenic changes [4]. Models describing the chain of events in detail leading to a transmission of known viruses can be applied to unknown pathogens in theoretical emergence events to better understand disease spread [5]. Systematic long-term monitoring of pathogen circulation and interspecies interactions may provide valuable insight on predictive trends or risk factors for disease emergence events.

#### Crowdsourcing and private funding

Over the next decade, we project a continued increase in the percentage of research funding from sources outside of government agency expert-directed and peer-reviewed grant programs. Crowdfunding for research and implementation projects provides opportunities for efforts that may not fit government agency priorities but appeal to members of civil society [6]. It allows for innovation by supporting new researchers and new ideas. It also challenges scientists to explain their efforts and anticipated research outcome in terms that are more easily understood by the general public, thus stimulating more public engagement in the process of science.

#### Mass mortality events

In recent decades, several sudden disease-driven mass mortality events have made conservation scientists take seriously into account the health aspect of conservation. The emergence of deadly diseases and infections has highlighted the need to deeply analyze these events, as the drivers prove to be multifactorial, having complex webs of causation that require profound and dedicated investigation. In developing countries, wildlife mortality events are rarely pursued past standard outbreak investigative procedures. This lack of proper response and deep investigation is due to limited capacity and resources, and in some cases, corruption at the government level. This problem constitutes a major challenge if we are to understand wildlife health in developing countries, and its implications in One Health at a global level.

#### Economic optimum for land development

Land use change is a leading driver of ecosystem degradation and desertification, biodiversity loss, contamination of clean water sources, climate change, and infectious disease emergence [7]. As global population and urbanization rates rise, the potential for negative human health consequences from land use change increases. Programs and projects designed to find the economic optimum of land development and create flexible, predictive models are vital to engage stakeholders to make safe and educated long-term policy decisions that maximize both public and private benefits. Making informed decisions on land use change can drastically reduce the negative economic impact on the local community, and avoid crises such as outbreaks of endemic diseases, and other small-scale epidemics.

#### Financial risk-rating instruments for disease outbreaks

The economic consequences of outbreaks extend far beyond the health sector, with recent epidemics inflicting significant losses on travel, tourism, extractive industries, agriculture, education, and more [8]. Innovative risk-based financing mechanisms are increasingly being pursued to avoid potential catastrophic losses associated with pandemics. The creation of pandemic insurance markets, while providing a mechanism to mobilize resources for containment and recovery, at present seeks to assist countries in their response and recovery, but lack incentives for prevention. If recent trends continue, the pace of disease emergence and spread threatens to make response efforts and insurance payouts cost prohibitive.

### HIGHLIGHT: changing the future of epidemic response

*Patrick Drury, Khristeen Umali, Renee Christensen, Sameera Suri - The Global Outbreak Alert and Response Network, World Health Organization*

This highlight presents an overview of the activities and future direction of a key WHO program that pools human and technical resources for rapid identification, confirmation and response to outbreaks of international importance.

The Global Outbreak Alert and Response Network (GOARN) is a network of over 240 public health institutions, international organizations, non-governmental organizations, UN Agencies, and disease specific, and technical networks. GOARN is an integral part of the WHO Health Emergencies Program (WHE), providing a mechanism to coordination international technical assistance; and to add direct technical assistance and operational capabilities to WHO’s traditional technical and normative roles.

WHE works with countries and partners to prepare for, prevent, respond to and recover from all-hazards that create health emergencies, including disease outbreaks, conflicts, and disasters. WHO leads and coordinates the international health response to contain disease outbreaks and provide effective relief and recovery to affected people.

The Health Emergency Program is built on timely, all-hazard alert, risk assessment and response to every significant new acute public health event around the world. Strengthening partnership with GOARN, the Global Health Cluster, Emergency Medical Teams; and Stand-by partners is essential for comprehensive assessment and planning of response, and for coordinated and predictable collective action, and support to countries and affected communities and populations. The WHO Health Emergency Programme also focuses on national prevention and preparedness plans and capabilities, and high-consequence disease-specific prevention and control strategies. Finally, WHE incorporates implementation of the WHO country business model in high-risk countries with protracted emergencies.

#### Active daily, worldwide

On a daily basis, all regions of WHO and headquarters are involved in global event-based surveillance using formal and informal sources of information. The same system is used worldwide, whether in Manila in the Western Pacific, Washington DC for the Pan-American Health Organization, Brazzaville for sub-Saharan Africa, and the other regional offices. This process of event-based surveillance includes working closely with the Food and Agriculture Organization (FAO) of the United Nations, and the World Organization for Animal Health (OIE) on zoonosis and acute emerging threats and disease outbreaks. Daily work involves verifying acute events and offering technical assistance and support. In this respect, WHO relies on GOARN partners for international coordinate, and to deploy response teams; while partners rely on WHO for leadership, information, logistics and security.

#### GOARN at a glance

GOARN was established in April 2000 with a founding group of 60 institutions and has grown to currently comprise a network of 248 institutions worldwide. It’s guided by a 21-member Steering Committee (SCOM) and Working Groups. WHO acts as an Operational Support Team and a Secretariat for GOARN.

The aims of GOARN are to coordinate rapid international support teams, provide assistance to countries to investigate and characterize events, assess risks, strengthen outbreak response, and support national outbreak preparedness.

**Fig. 1:**
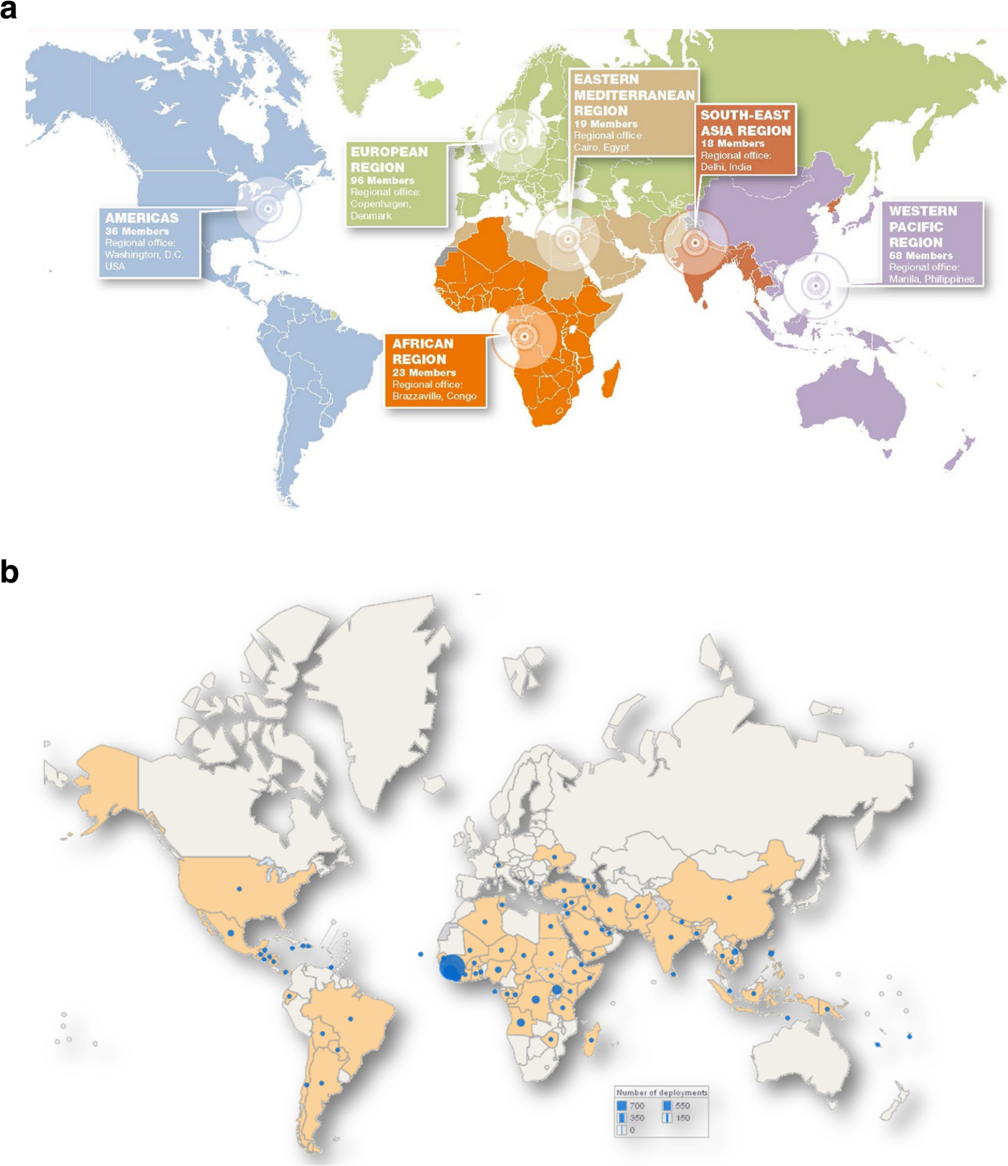
**a** and **b** Between 2000 and 2018, GOARN deployed 134 field missions in 87 countries. This translates to approximately 2787 field deployments or 82,359 person-days in the field

GOARN’s partners and networks work in a global and regional approach (Fig. 1a-b):

The two most recent deployments are both in Africa. One is in response to the Ebola outbreak in the Equateur Province of the Democratic Republic of the Congo (DRC). Two hundred and eighty-five staff are deployed by WHO in the field - of whom, 37 are directly from GOARN partners. The other response is for the major Listeria outbreak in South Africa, where two GOARN staff were deployed to support and working with local agencies and partners to identify the specific food production site as the source of outbreak.

#### GOARN moves into the future

As part of its review process, the WHO Global Health Crises Task Force remarked that robust security systems are critical for GOARN operations and for a strong WHO technical, logistics and operational platform capable of supporting countries and coordinating international response. It was recommended that WHO should strengthen its partnership with GOARN to enhance surveillance (alert and early warning), risk assessment and risk communication, and response. Moreover, WHO should encourage long-term investments in partners, to ensure increased standing response capacity; and in the strategic development of GOARN, to integrated national, regional and global capabilities; and build and integrate a network-of-networks providing capacity and rapid response for the health operations and research in the field – including social sciences, risk communication and community engagement, field epidemiology, operational analysis and planning, laboratory capacity, infection prevention and control, and case management. In turn, GOARN should support the WHO Health Emergencies Program with technical and operational innovation, and networking that will drive effective preparation, alert and response to public health emergencies.

As a result, the GOARN Steering Committee came up with a set of ideas and plans to ensure that the network remains fit for purpose going forward. All these plans have been endorsed by the whole network, with a key recommendation among partners being to “think One Health” and engage partners and stakeholders accordingly.

A first requirement is for GOARN to be engaged more actively to support national institutions in alert, risk assessment, and response planning. This means refocusing the constituency of GOARN by directly involving in-country technical agencies and partners that are dealing with any particular event, as well as the regional and global stakeholders that can provide additional capacity and support, if requested. One of the ways this is being put into practice is via a weekly teleconference to coordination on acute events which includes FAO and OIE and the appropriate local partner. For example, in responding to the current outbreak of Ebola in Equateur, this includes regional partners, and when possible national agencies. Likewise, during the recent Lassa fever outbreak in Nigeria, the Nigerian Centre for Disease Control (NCDC) lead the response planning and participated actively with international partners. For the listeria outbreak in South Africa, the National Institute for Communicable Diseases (NICD) South Africa – a long standing partner in GOARN – provided technical information and kept international partners abreast of development in the outbreak, the focus of investigations, and response.

A further area for development is the building of rapid response capacities and teams. With so many events and the potential for any one of them to accelerate out of control, WHO and GOARN face great pressure to be able to identify very rapidly the core expertise – which often needs to be individually tailored – that can be deployed in a highly scalable way.

Training remains essential, and this will include outbreak response scenario-based training. Twenty-seven institutions are already working on the development of a tiered training program and its translation into different languages. This is a significant source of additional capacity that is available for field deployment.

Communications also needs to be firmly embedded in the network’s approach, enabling outbreaks of potential international concern to be identified; public health information to be communicated; international risk assessment to be offered; and direct technical and operational assistance to the affected country to be provided.

#### GOARN 2.0 in a nutshell

In short, GOARN 2.0 involves an expanded and intensified involvement of partners in a range of activities, and possible engagement with new partners from public, private and civil society. The Steering Committee will provide leadership on collaboration and strengthen the network by attracting and retaining the best institutions. Partners and the Operational Support Team need to be well-resourced, and it’s essential to advance this shared vision and consensus on the priorities of GOARN.

### HIGHLIGHT: empowering communities for the containment of infectious outbreaks

*Christophe Longuet, CORDS*

The Connecting Organization for Regional Diseases Surveillance (CORDS) is a non-profit NGO located in Lyon, France. It was founded in December 2007 at a meeting of Public Health Surveillance Networks at the Rockefeller Bellagio Center in Italy, where representatives of the networks were gathering to share lessons learned, best practices and challenges.

CORDS receives funding from the Rockefeller Foundation, Skoll Global Threats Fund, the Bill & Melinda Gates Foundation, NTI, Fondation Mérieux, Search for Common Ground, the Canadian Ministry of Foreign Affairs and the West African Health Organization.

CORDS has three strategic pillars: to promote innovation, improve capacity and build sustainable networks, all within the One Health framework. Its overall mission is to catalyze exchange and collaboration amongst regional disease surveillance networks, throughout the world, to strengthen their capacity to detect and control the spread of epidemics. This is translated into practice by pursuing four key actions:

- Further strengthening national capacity and regional networks based on effective communication through electronic means, regular meetings and joint projects
- Promoting and enhancing the overall global capacity for infectious disease surveillance by connecting its regional networks into a global cooperative activity
- Developing and encouraging collaboration between the human, animal and agricultural sectors to achieve a holistic approach to infectious disease surveillance
- Promoting the development of national capacities and new regional networks, particularly in Africa and South Asia.

**Fig. 2:**
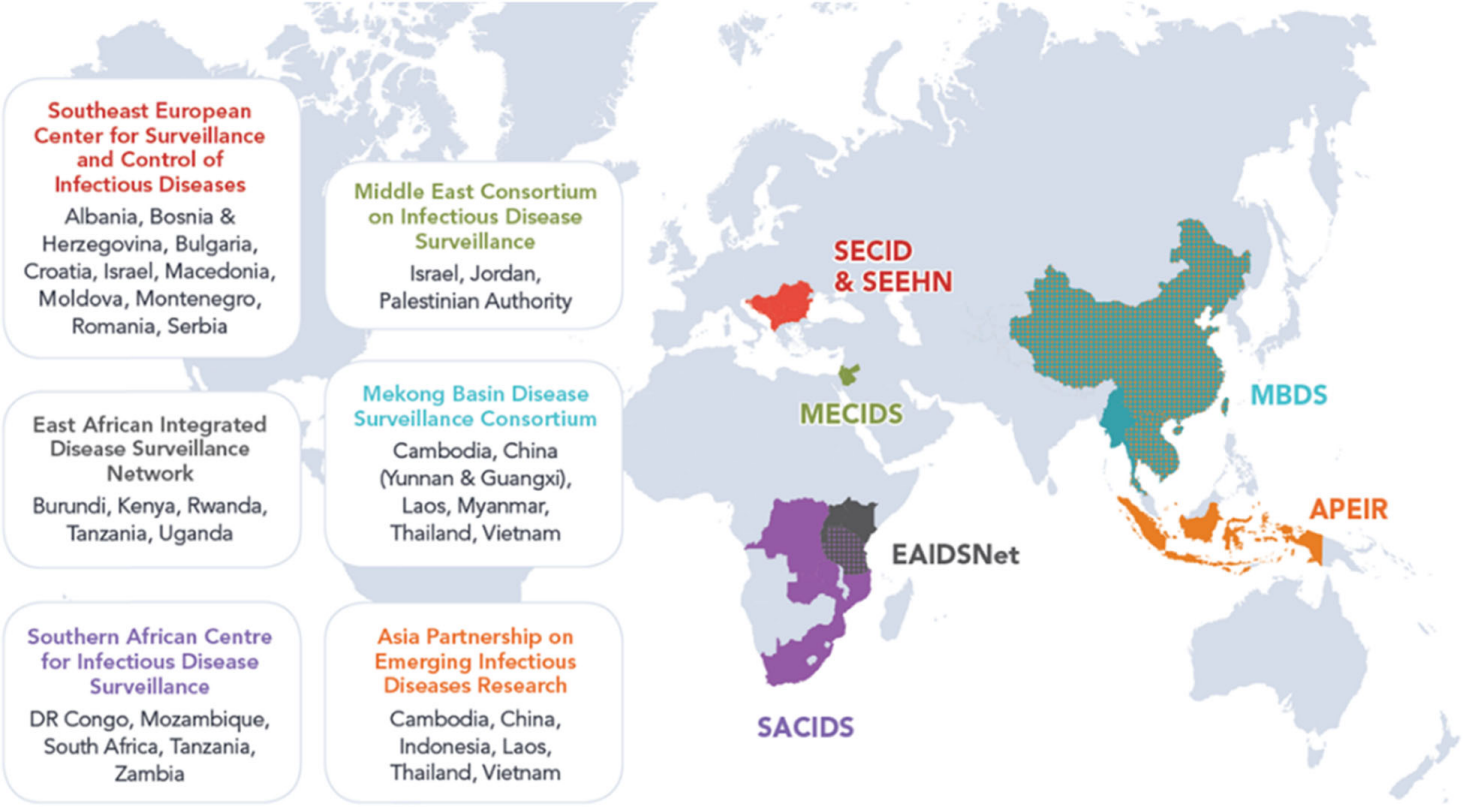
The CORDS’ member networks

CORDS is comprised of six regional networks working to detect and control the spread of infectious diseases by exchanging information between surveillance systems (Fig. 2):

#### The solution needs to be the community solution

CORDS has a clear focus on the community, and aims to ensure that communities are safe from the spread of infectious diseases in animals and humans, by containing outbreaks at source. To enable this, communities have to be empowered, so CORDS is keen to develop and offer new and innovative tools to better empower communities. For example, the use of participatory surveillance tools can help communities make informed decisions about their health, with links between primary, secondary and tertiary levels of care.

CORDS also wants to see communities working together closely across borders to prevent and control regional epidemics, and also believes that community-level needs can play a key role in informing global health policy.

#### Four innovative projects at community level

In the CORDS’ MECIDS network (Middle East Consortium on Infectious Disease Surveillance), a seroepidemiological study was conducted to identify the transmission in Jordan, Israel and Palestinian authorities of the **Middle East Respiratory Syndrome coronavirus (MERS-coV)**. Current scientific evidence suggests that dromedary camels are a major reservoir host for MERS-CoV and an animal source of MERS infection in humans.

The study concluded that despite the mass gathering and extremely intensive population “mixing”, pilgrimage to Mecca was not associated with an increased risk of MERS-CoV acquisition. No subclinical infection was detected in samples from the general population of camel owners. And no camel to human transmission was identified by screening with Euroimmun ELISA (Prof. Dany Cohen, MECIDS, unpublished report). However, studies are still underway with more sensitive and specific assays at the Erasmus MC Laboratory Rotterdam, the Netherlands, and further studies are needed to further examine transmission of MERS-coV in the region.

Also via MECIDS, in Albania, Jordan and Pakistan, a **Leishmaniasis gap analysis** was conducted. Leishmaniasis is a neglected disease, in that it affects neglected or marginalized peoples, but is the second most prevalent parasitic infection in the world after malaria. Jordan is a hot spot for Leishmaniasis, with increased cases due to the influx of Syrian refugees. The investigation covered the status of Leishmaniasis and the current surveillance, treatment and control activities in each country, with a critical evaluation of the effectiveness of these activities.

A number of common findings were published (Dr. S. Bino SECIDS, Dr. K. Kanani, MECIDS, Dr. S. M. Mursalin, POHA, unpublished reports). No population control measures were in place and no active surveillance was carried out in dogs, nor was there any vector control program dedicated for sand flies. Case detection and clinical management protocols were outdated, while there was only intermittent or a lack of medication in public hospitals and a lack of diagnostic tests. In short, the capacity for early disease detection at regional or district levels was low, and there was also a low community perception and a lack of community programs, especially in poor areas. The recommendation was to have Integrated One Health surveillance incorporating case management, vector control and animal reservoir control.

The APEIR network of CORDS (Asia Partnership on Emerging Infectious Diseases Research), with support from the International Development Research Centre (IDRC) Canada, implemented a 5-year study to explore, develop, and assess the effectiveness of a strategy for the **proper use of antimicrobials in humans and animals to control antimicrobial resistance** in Indonesia, Thailand, Vietnam, Laos and some regions of China (Prof. A. Soebandrio, APEIR, unpublished report).

During these 5 years, baseline surveys were conducted of antibiotic use and access, along with an *E. coli* sensitivity survey in human, animal and environmental isolates. The public health and animal health workforces in these countries were also trained.

A further project is being conducted in Tanzania by the SACIDS (Southern African Centre for Infectious Disease Surveillance) network of CORDS. Afyadata is a tool that analyzes data collected from the field and intelligently sends feedback to the data collector and alerts higher authority officials if any abnormal pattern is discovered in the data collected. It aims to **empower the community to perform animal and human health surveillance** with simple tools that they own. With this tool, communities have been able to rapidly provide alerts and consequently receive fast response, enabling them to protect their animals and population.

From March to May 2017, a total of 212 livestock cases and 10 human cases were reported by Community Health Reporters using Afyadata in the Ngorongoro and Morogoro districts. The tool has helped communities learn a lot about what kinds of diseases are in their locality and how to manage them (Prof. M. Rweyemamu, SACIDS, unpublished report).

#### CORDS’ messages for policymakers

Infectious disease outbreaks that pose a threat to the public health often emerge from close contact between animals – domestic or wild – and humans, in underserved regions. They can swiftly cross borders without being identified, particularly in countries with poor surveillance systems. Efforts to stop their spread must start at “the first mile”, where communities should be empowered to participate in early identification of, and response to, emerging infectious diseases in a One Health approach.

It’s therefore vital to think and act at community level to achieve Global Health Security. This means understanding the needs of communities and providing them with sustainable benefits and acceptable solutions. Health also extends beyond physical, psychological and social well-being; “economical health” is also important, for example by ensuring that laws that enable farmers to seek compensation for the loss of livestock are enforced.

Strengthening health systems for humans and animals in underserved regions is key. In this respect it’s important to have national laboratory and national public health institutions not only in existence, but closely connected to the communities and the care system to enable outbreaks to be identified. Also essential is to build excellent surveillance and care systems for humans and animals.

Finally, communication is key, which means strengthening relationships with the underserved communities, as well as with scientists, decision-makers – in health, agriculture and other sectors – and between regions, as challenges faced in different parts of the world are often similar. Solutions identified in some regions can therefore be usefully applied in other countries and continents. CORDS therefore proposes South-South-North collaborations based on trust at community, cross-border and cross-region levels.

### ACTION: 1st international one health forum, November 2019, Africa

Recent global disease events have highlighted the impact of zoonoses on human and animal health. It is clear that cooperative solutions are needed to address biological threats and thus global health security. Zoonotic diseases account for more than one billion cases and a million deaths per year, according to the World Bank. Public health systems will only grow stronger by applying a holistic view on the health of humans, animals and the environment, thus calling for a One Health approach. In addition, we have entered a new era of (re-)emerging and recurring global health threats that call for a longer-term, more strategic approach to global health security.

Across the African continent, initiatives have already been set up. Thus, meetings are being organised, and networks established that adopt the One Health approach, engaging public health practitioners, scientists, policy makers and workforce. Yet, these initiatives are not well connected nor integrated.

Established in January 2017, the Africa Centres for Disease Control and Prevention (Africa CDC) is a new public health agency of the African Union. Africa CDC seeks to make Africa safer and healthier by strengthening Africa’s health institutions, particularly its national public health institutes (NPHIs). One Health presents a challenge for NPHIs, because there is no Africa-wide consensus for the public health practice of One Health, including surveillance and disease control. For surveillance, critical questions include:

- what diseases should NPHIs focus on?
- how should NPHIs conduct surveillance for those diseases in the human health sector?
- what tools should NPHIs use to assess risk, inclusion thresholds for investigation and control in collaboration with the animal health sector?

The International One Health Forum focussed on strengthening the public health practice of NPHIs in Africa by convening representatives from existing One Health initiatives and networks and having them present their recommendations for best practices regarding zoonotic disease surveillance. Substantial work on building consensus for One Health public health practice has already been developed by WHO, FAO, OIE, the U.S. Centers for Disease Control and Prevention, and other partners. In addition, many African countries have conducted disease prioritization workshops and initiated programs for surveillance and control of specific zoonotic diseases. While NPHIs serve the human health sector, the Forum ensured adequate representation from animal and environmental sectors to ensure that recommendations for prioritization, surveillance, and risk assessment include input from all relevant sectors.

Three questions were at the core of the 1st International One Health Forum:

1. What are the top zoonotic diseases of importance across the African continent?
2. How should NPHIs conduct surveillance for a selection or all of these priority zoonotic diseases?
3. How do NPHIs assess the risk of zoonotic cases and clusters in collaboration with animal and environmental sectors?

## Biological threats

### HIGHLIGHT: strengthening global biological security - a global perspective on biological threats and decision making

*Rebecca Katz PhD MPH, Georgetown University Medical Center, Washington D.C., USA*

An overview of global biological threats, who are the actors deciding how to address these threats, and on what data are these decisions based?

#### Biological threats

Emerging infectious diseases are increasing in number. We see examples every year, including the emergence of new coronaviruses (https://www.nih.gov/news-events/news-releases/new-coronavirus-emerges-bats-china-devastates-young-swine). New studies estimate that there are over 1 million viruses in mammals, and approximately half of those have the potential to threaten human health. At the same time, risk factors are increasing. The world is more interconnected than ever before, with closer inter-relationships between humans, animals and environmental health. Climate change, population growth, urbanization and human migration all contribute to the likelihood of biological threats.

Never too far away from the news headlines is the deliberate use of biological agents, or the geopolitical shifts that can lead to the possibility of state or non-state actor use of biological weapons in conflicts. And, advances in science and technology are capable of both positively impacting the ways we investigate and respond to biological threats, as well as contributing to the biological threat in the first place.

#### How do we think about policy and response to biological threats?

**Fig. 3:**
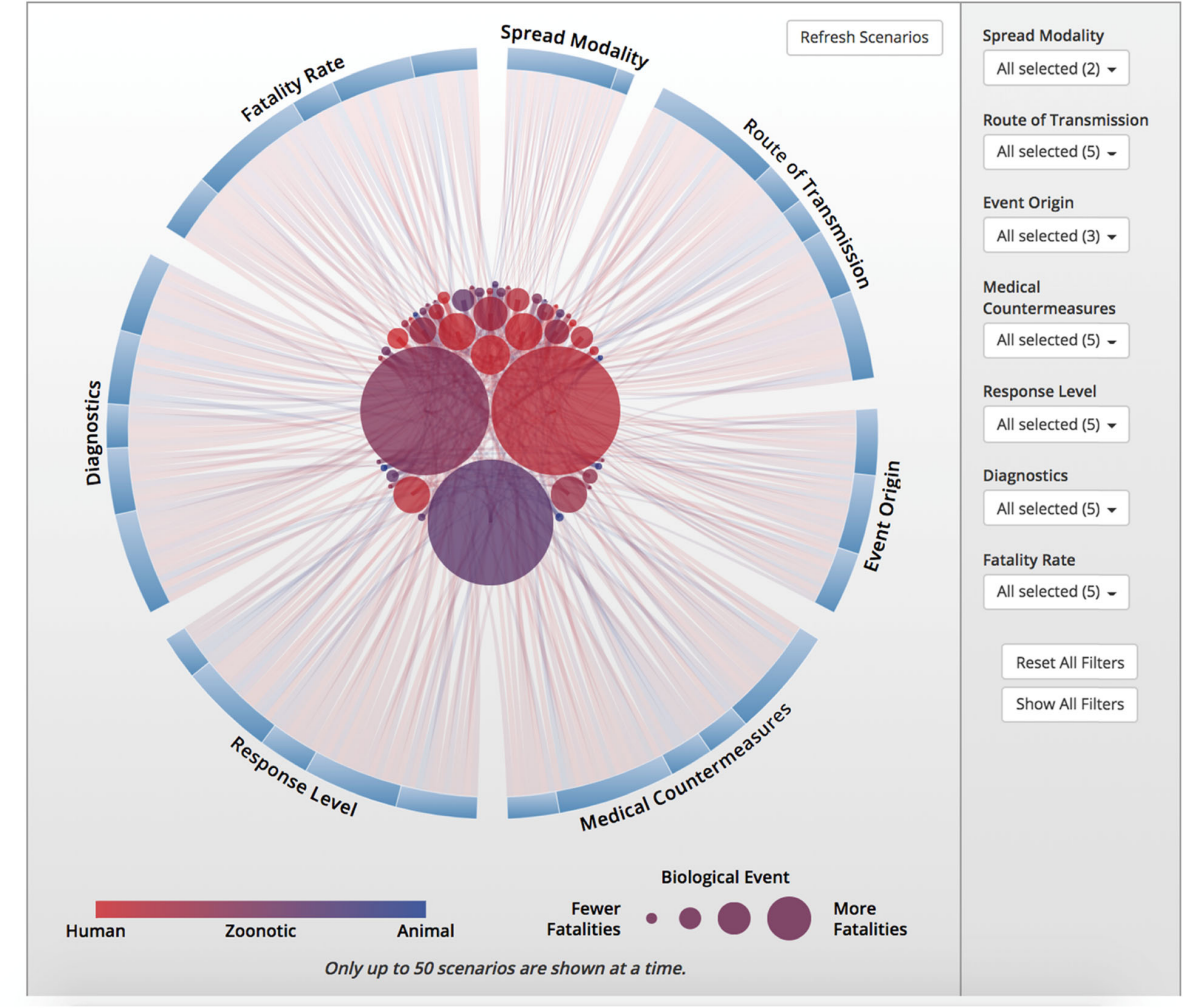
Response planning must be able to adapt to an increasingly broad range of scenarios. Available at: https://bioscenarios.talusanalytics.com

Preparing for and responding to biological events is complicated by the fact that no two events are the same. To demonstrate this complexity, we developed a framework to categorize biological events based on the 12 characteristics that drive planning and response decision-making (such as event origin, type, route of transmission, outbreak location, response level etc.). It generated approximately 22 million notional events (Fig. 3), which clearly demonstrates just how diverse the biological threat is, as well as how response planning must be able to adapt to an increasingly broad range of scenarios (https://bioscenarios.talusanalytics.com/).

Preparedness though, however complicated, is essential. At the Munich Security Conference in February 2017, Bill Gates and his team of modelers indicated that the threat of a large-scale pandemic is very real: “A fast­moving airborne pathogen could kill more than 30 million people in less than a year … and there is a reasonable probability the world will experience such an outbreak in the next 10-15 years”.

It is frightening to talk about a catastrophic pandemic, but also challenging to engage decision makers in low probability events. The World Health Organization (WHO), however, screens approximately 3000 signals a month, follows up on 300 of these and closely investigates 30 per month, meaning that there are very real biological threats happening every day. Recent outbreaks include Ebola in the Democratic Republic of the Congo, HIV cases in Pakistan, Dengue fever on Réunion Island, and MERS-CoV in Saudi Arabia (https://www.who.int/csr/don/en/).

The economic burden of biological threats will undoubtedly be enormous. Experts estimate that a global influenza pandemic could cost USD 570 billion [9]. This isn’t too far short of the potential economic impact of climate change, which has been estimated at USD 890 billion.

#### Who is addressing these threats?

**Fig. 4:**
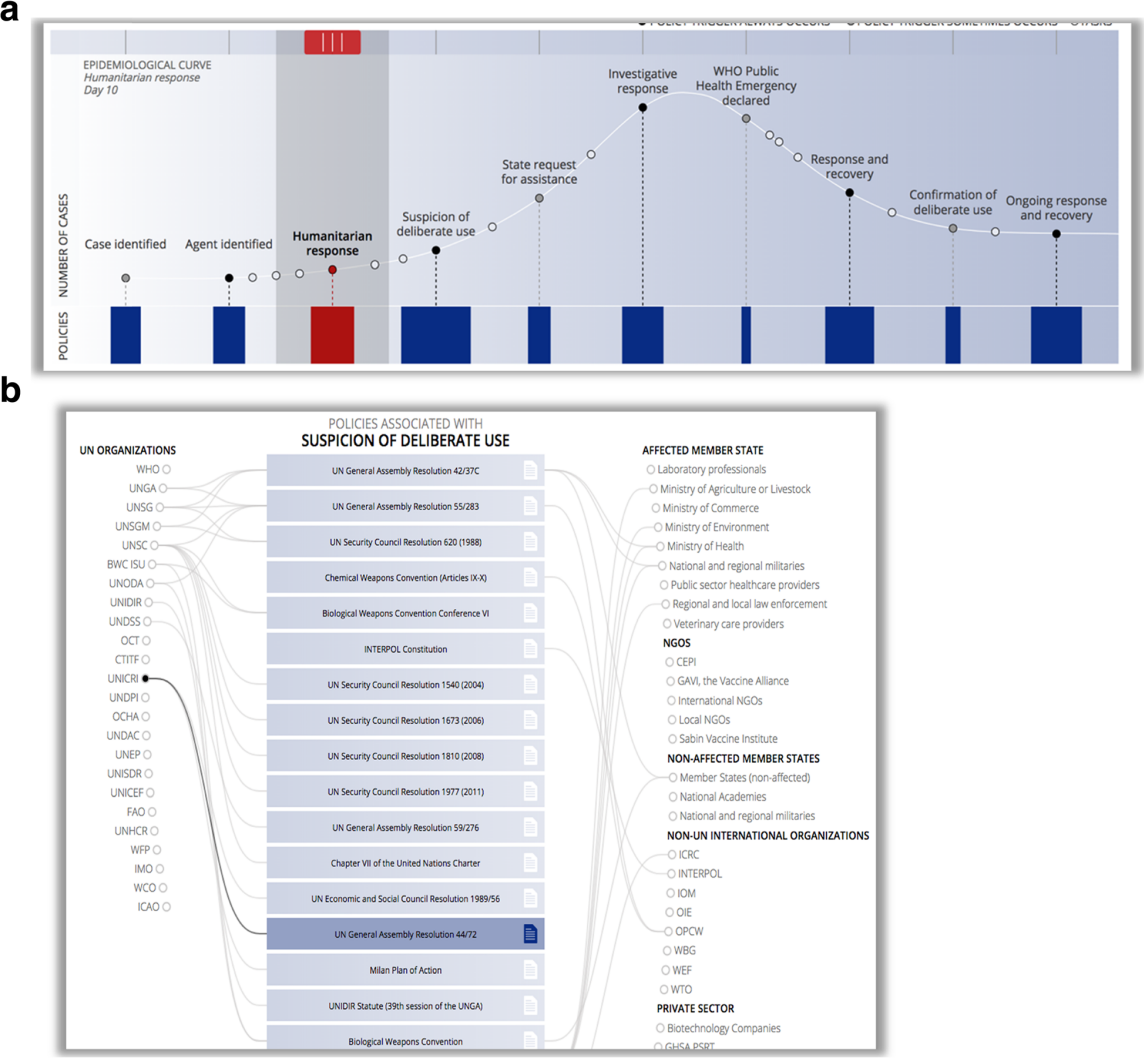
**a** and **b** Epidemiological curve and associated policies when a biological threat is potentially deliberate. Available at: dbe.talusanalytics.com

There is no easy answer to this question, because addressing biological threats involves many activities, from preparedness, detection, reporting, response, recovery, investigation to governance. All these activities require experts in a vast number of fields; just a short list includes epidemiology, lab diagnostics, risk communication, emergency management, clinical medicine, logistics, operations & leadership, command & control, microbial forensics, data mining, anthropology, social science, and media relations. And when a biological threat is potentially deliberate, identifying all the relevant stakeholders involved in addressing and responding to the threat becomes even more complex (Fig. 4) (https://dbe.talusanalytics.com/).

***Frameworks for biological threats*** are diverse, ranging from local implementation guidelines through national policies, strategies and regulations up to regional and ultimately international agreements. Coordination is provided by international organizations such as the World Organization for Animal Health (OIE), the Food and Agriculture Organization (FAO) of the United Nations, and the World Health Organization (WHO), along with multilateral groups such as the Global Health Security Agenda (GHSA) and the Global Partnership Program.

A useful way to consider the ***drivers of decisions regarding biological threats*** is the traditional hierarchy of foreign policy governance functions developed, described by David Fidler in 2006 [10], with high politics at the top and soft power at the bottom: public health normally resides in the low part of the spectrum. However, what’s noteworthy is that over the past decade, addressing global health – and biological threats in particular – has become part of the high foreign policy functions, with an increasing emphasis on global health security. It’s important to recognize that it’s the security component that really drives engagement by foreign policy functions of government.

Another major driver is resources. Unprecedented levels of resources are being channeled into global health activities. For example, the Bill and Melinda Gates Foundation now stands alongside UN agencies, the World Bank and bilateral development agencies as the leading contributors to global health. Since 1990 there has been a quadrupling in global health expenditures. This trend is also echoed in voluntary contributions; over 70% of all funding to WHO comes from these sources.

As to the *global health actors who are addressing biological threats,* they come from both public and private sectors. This is a rapidly evolving field – and consequently increasingly crowded – incorporating public-private partnerships, international finance institutions, trade organizations, self-empowered individuals, philanthropic capitalists, non-governmental organizations and civil society.

#### What evidence is used to make decisions?

When it comes to making decisions on biological threats, it’s important to consider what evidence is being used to inform actions. Possible sources of this evidence are academic papers, public opinion, social media, conference presentations and internal data analytics. Often, however, we must admit that sometimes the factors that contribute to decision making is linked to whomever the loudest or most powerful person in the room is, recognizing that power might come from a variety of sources.

There are, however, extensive efforts by the international community to generate data that can be used as evidence to support actions. The Joint External Evaluation (JEE) of the WHO is a voluntary, collaborative, multisectoral process to assess the ability and capacity of countries to prevent, detect and rapidly respond to biological threats. It helps countries identify the most critical gaps within their public health systems and then prioritize opportunities for preparedness and response.

Drawing from and complementing the JEE are efforts such as the forthcoming Global Health Security Index which will provide a public benchmarking of global health security conditions and compliance with international standards for epidemic preparedness. Additionally, coalitions are being created to monitor and evaluate efforts and mechanisms at country and global levels to address biological threats. Such initiatives aim to ensure that the key decisions on biological threats are being made by the right actors, based on the right data.

#### HIGHLIGHT: biological threat reduction strategies

*Keith Hamilton, World Organisation for Animal Health (OIE)*

This highlight provides an overview of some of the biological threat reduction strategies being implemented throughout the world.

There are many ways to define a biological threat (biothreat). Merriam-Webster defines it as a threat posed by a harmful biological agent, while the US Department of Defense calls it a threat that consists of biological material to be deployed to produce casualties in personnel or animals or damage plants. At the World Organisation for Animal Health (OIE), we define a biothreat as “the accidental or deliberate release of a pathogen or toxin into a susceptible population.” This could include biocrime, biological warfare, bioterrorism, agroterrorism and agrocrime, depending on the perpetrator and the target.

**Fig. 5:**
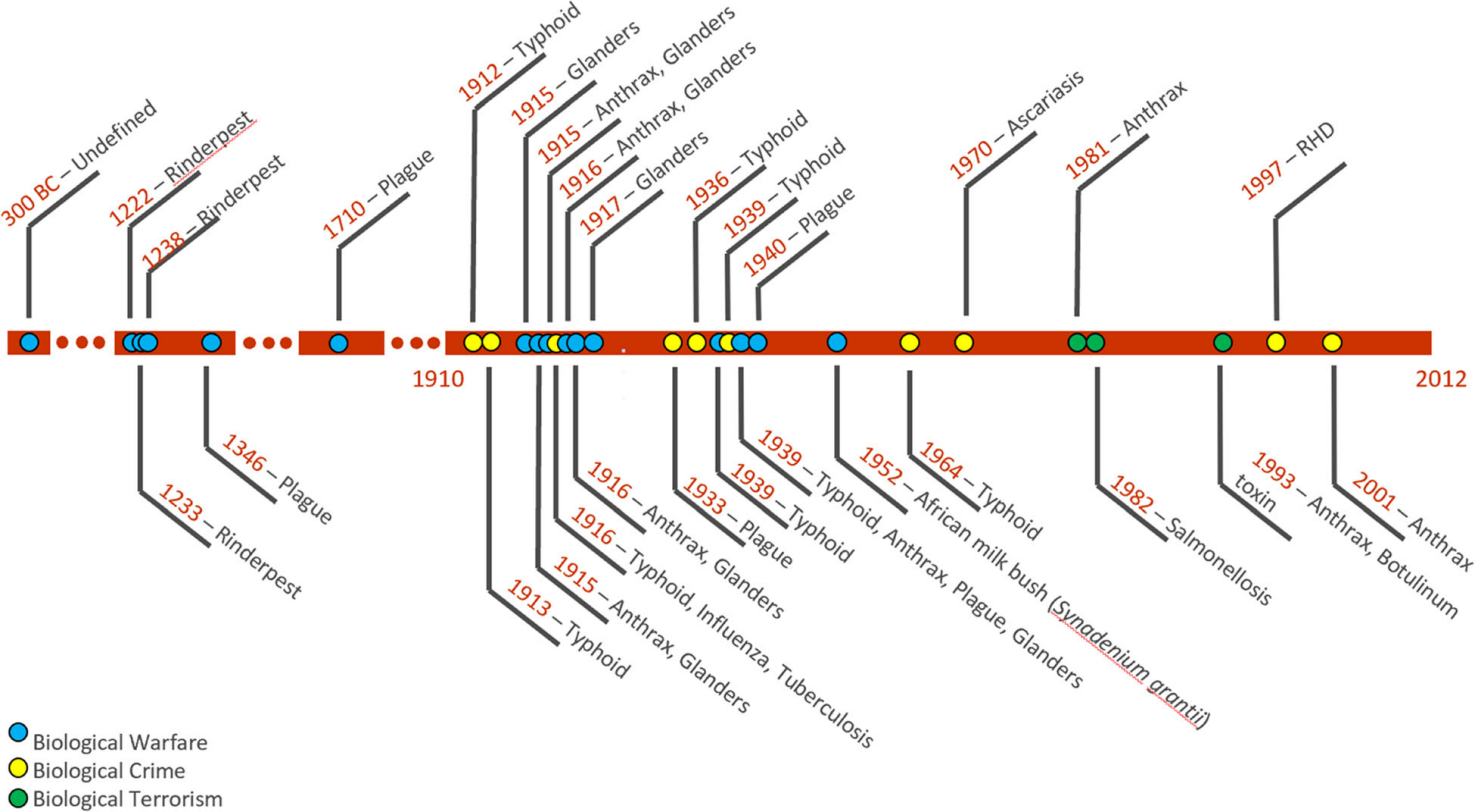
Timeline showing confirmed use of animal pathogens or toxins used in biological warfare, biocrime or bioterrorism through the centuries (Developed by Keith Hamilton and Bruno Bastos)

The timeline below (Fig. 5) displays confirmed use of animal pathogens or toxins used in biological warfare, biocrime or bioterrorism through the centuries, and clearly highlights that the threats are real, and are not going away. Because the capability to synthesize and engineer genes is becoming more widely available, and can be done at lower cost, threats from the misuse of pathogens may be increasing.

Moreover, biological threats in whatever form have huge impacts on human life, national and global economies, the supply of food, and civil stability. These impacts are often connected. For example, it does not take long for a food shortage to lead to civil instability. The quote along the lines of ‘any society is only three meals from a revolution’ has been attributed to numerous leaders and philosophers.

#### The health-security intersection

When it comes to the development and implementation of strategies and activities surrounding biological threats, the health and security sectors share a number of common interests. These include a commitment to peace, stability and prosperity; strong mechanisms to prevent, detect and respond to disease outbreaks; sustainable, safe, and secure laboratories; responsible scientists; and a global approach. Cooperative threat reduction is built around these common interests.

Biothreat reduction strategies can and do address different levels: global, societal, national, field and laboratory. Some strategies work at all levels, while others work across just one level, indicating that in real life, biological threat reduction strategies are a complex mix of horizontal, top-down and bottom-up approaches. For maximum impact and effective use of resources these strategies should be well coordinated, synergistic, and adapted to the local context.

#### The cooperative threat reduction program

The Cooperative Threat Reduction (CTR) Program, also known as the Nunn-Lugar Act after the work done by US senators Sam Nunn and Richard Lugar, was created in 1986 and is a good example of a biothreat reduction strategy. It was established for the purpose of securing and dismantling weapons of mass destruction and their associated infrastructure in the former states of the Soviet Union. It was also a response to knowledge that biothreat material existed across the USSR in different locations, and therefore focused on securing those materials and making sure they didn’t fall into the wrong hands such as rogue states or terrorists, hence it was described as “proliferation in reverse.”

The program looked into nuclear, chemical and biological threats. The first two threats involve dismantling equipment and warheads; biological threat reduction is a little more complicated as it also involves engaging scientists to make sure they are not tempted to be employed by other countries or groups intent on developing biological weapons. It also involves securing pathogens and making sure they are held in high containment facilities.

The objectives of the CTR Program – which align very closely with the aims of the health sector on this topic – are to consolidate and secure dangerous pathogen collections into central reference labs or repositories; to improve the safety and security of biological facilities; to enhance partner states’ capabilities to detect, diagnose, and report bio-terror attacks and potential pandemics; and to engage scientists with biological weapon-related expertise in research and careers that support peaceful objectives such as health protection, medical countermeasures, diagnostics.

#### Global partnership against the spread of weapons and materials of mass destruction

Known in short as the Global Partnership (GP), this is a different type of strategy that works at a truly international level. It’s a 31-member international initiative that aims to prevent the proliferation of chemical, biological, radiological, and nuclear (CBRN) weapons and related materials. The European Union is one of the 31 members, which in effect adds another 28 countries to the membership list. A number of organizations such as OIE, WHO, FAO and Interpol participate as observers. The Global Partnership was established at the 2002 G8 Kananaskis summit.

In practice, the GP operates through several working groups that address specific threats. These groups agree on deliverables which they can all work towards as a cooperative. One such working group is the GP Biosecurity Sub-Working Group (BSWG). It has stipulated a number of deliverables, many of which are very closely aligned to the Cooperative Threat Reduction Program described earlier:

1. Secure and account for materials that represent biological proliferation risks
2. Develop and maintain appropriate and effective measures to prevent, prepare for, and respond to the deliberate misuse of biological agents
3. Strengthen national and global networks to rapidly identify, confirm and respond to biological attacks
4. Reinforce and strengthen biological non-proliferation principles, practices and instruments
5. Reduce proliferation risks through the advancement and promotion of safe and responsible conduct in the biological sciences.

#### OIE biological threat reduction strategy

The OIE understands the importance of working with the health and security sectors and is committed to a world that is safe and secure from the accidental or deliberate release of animal pathogens, including zoonoses. Its biological threat reduction strategy aligns with its 6th strategic plan and falls under the umbrella of risk management. It is based on the OIE philosophy of solidarity and cooperation and adheres to the principle that the same mechanisms protect against natural, accidental and deliberate releases.

The focus of the OIE strategy is on strengthening, enhancing, and developing cross-links between existing health systems. Moreover, OIE sees the importance of engaging its membership in any new strategy and is therefore transparent about the objectives of the strategy and its implementation, all of which are shared with its members. OIE has also held two global conferences on biological threat reduction, to which OIE’s members and partners were invited to attend and provide their input.

The OIE Biological Threat Reduction Strategy has delivered a number of concrete outputs. OIE now operates a Collaborating Centre on biological threat reduction, which provide training and technical support to countries throughout the world. The OIE, jointly with FAO, has implemented a post rinderpest eradication strategy which has led to the sequestration and destruction of rinderpest virus material to avoid a laboratory accident or deliberate release; approval of several safe and secure rinderpest holding facilities to store remaining stocks of virus; and a ban on research involving the handling or manipulation of rinderpest virus, unless approved by OIE and FAO. The OIE has also worked on strengthening surveillance networks that can detect the possibility of deliberate release of biological agents (as well as natural disease outbreaks) and has developed guidance for its member countries on how to investigate suspicious animal or health events which might indicate some nefarious activity. This also involves engaging with the law enforcement sector.

A number of disease outbreak simulation exercises have been held to test the response of countries to disease outbreaks, whether they have been natural or deliberate. OIE has also worked to support and strengthen veterinary services (and legislation) to better comply with OIE’s standards.

Experience has shown that the sustainability of capacity building efforts is a real challenge.

Recently, a sustainable laboratories initiative has been launched in collaboration with Chatham House. This initiative aims to better understand the determinants of laboratory sustainability and to identify solutions which will improve the sustainability of laboratories in the longer term, meeting both the needs of the health and security sectors. OIE also reaches out to and works with other agencies that share a common interest to tackle biological threats, such as Interpol, the Biological and Toxin Weapons Convention (BTWC) of the UN, the UN Security Council Resolution 1540 which deals with nuclear, chemical or biological weapons, and the UN Office for Disarmament Affairs (UNODA).

#### Advantages of cooperation at the health-security intersection

If both the health and security sectors succeed in addressing common interests, it’s a win-win situation. Conversely, if one sector fails, it’s a failure for both sectors. Cooperation of the health and security sectors is an efficient use of resources. It’s also a sustainable way to invest in mechanisms that respond both to day-to-day events as well as to unusual events such as intentional releases. Finally, synergy brings about increased effectiveness.

The health-security intersection is built on common goals and interests and provides concrete outputs that benefit both the health and security sectors. However, with such a hierarchy (or possibly a heterarchy) of strategies to address biological threat reduction, the need for in-depth coordination among all parties is paramount.

### HIGHLIGHT: global health security and the future of biological threat reduction

*Dr Maurizio Barbeschi, Sabri Gmach, John Hart, Jesse McLeay, Gaetano Morelli and Jan van Aken.*

*WHO, Health Emergencies Programme*

Through its 13th General Programme of Work, 2019–2023, the World Health Organization (WHO) aims to ensure healthy lives and to promote well-being for all through achieving universal health coverage, addressing health emergencies and promoting healthier populations. These three interlinked strategic priorities are founded on the Sustainable Development Goals (SDGs) of the United Nations. In practice this means that at least 1 billion more people must obtain access to essential health services in each five-year period between 2015 and 2030, be better protected from health emergencies, and enjoy better health and well-being. These objectives are operationalized by utilizing the revised 2005 International Health Regulations (IHRs) and a health emergency preparedness strategic framework for the promotion of intersectoral public health security coordination.

#### Preparedness and response to disease outbreaks

A range of planning factors and sustainable capacity building are required to ensure effective preparedness and response to disease outbreaks. An epidemic typically involves human-to-human transmission, nosocomial transmission in health care facilities, or transmission from animals to humans (zoonosis).

A pandemic may be understood as “the globalization of pathogens”, which not surprisingly is exacerbated by global travel of people, animals and vectors, and global trade of animals and their products, vaccines, medical products.

Factors that contribute to epidemics may include urbanization, mass gatherings, and accidental or deliberate use of biological agents for malicious purposes. Detection of a deliberate use is more difficult in cases where the occurrence of a group of similar illnesses derived from a common source beyond what would normally be expected in a given community or region.

#### The role of the international health regulations

By undertaking to implement the IHRs, 196 countries, including all WHO Member States, have committed to strengthen global health security through the early detection of and effective response to events that pose a risk to public health. This requires the rapid identification of unusual public health events through effective national surveillance systems which link directly to the implementation of timely and appropriate responses.

The IHRs have two fundamental components: 1. internationally coordinated monitoring, information sharing & response; and 2. the strengthening of core public health capacities to detect, assess, respond and recover in every single country, including at points-of-entry. The regulations do not distinguish between naturally occurring, deliberate or accidental spread of pathogens.

#### Joint evaluation tool

WHO’s Joint External Evaluation (JEE) Tool is utilized to evaluate a country’s capacities for health security. WHO developed the voluntary tool in 2016 and revised it in 2018. The JEE’s 19 technical areas are grouped into the three core elements of prevention, detection, and response.^1^ Countries may use the JEEs to develop tailored national action plans against biological and chemical threats.

#### Is the world prepared?

Despite the work carried out within this framework and various other international initiatives and commitments, the question still has to be asked: Are we adequately prepared for public health emergencies, including the deliberate release of pathogens?

The IHR core capacities are those required to detect, assess, notify and report events, and respond to public health risks and emergencies of national and international concern. The IHR monitoring process involves assessment of the development and implementation of core capacities at points-of-entry and for IHR-related hazards. These hazards may be biological, chemical, radio-nuclear in nature.

**Fig. 6:**
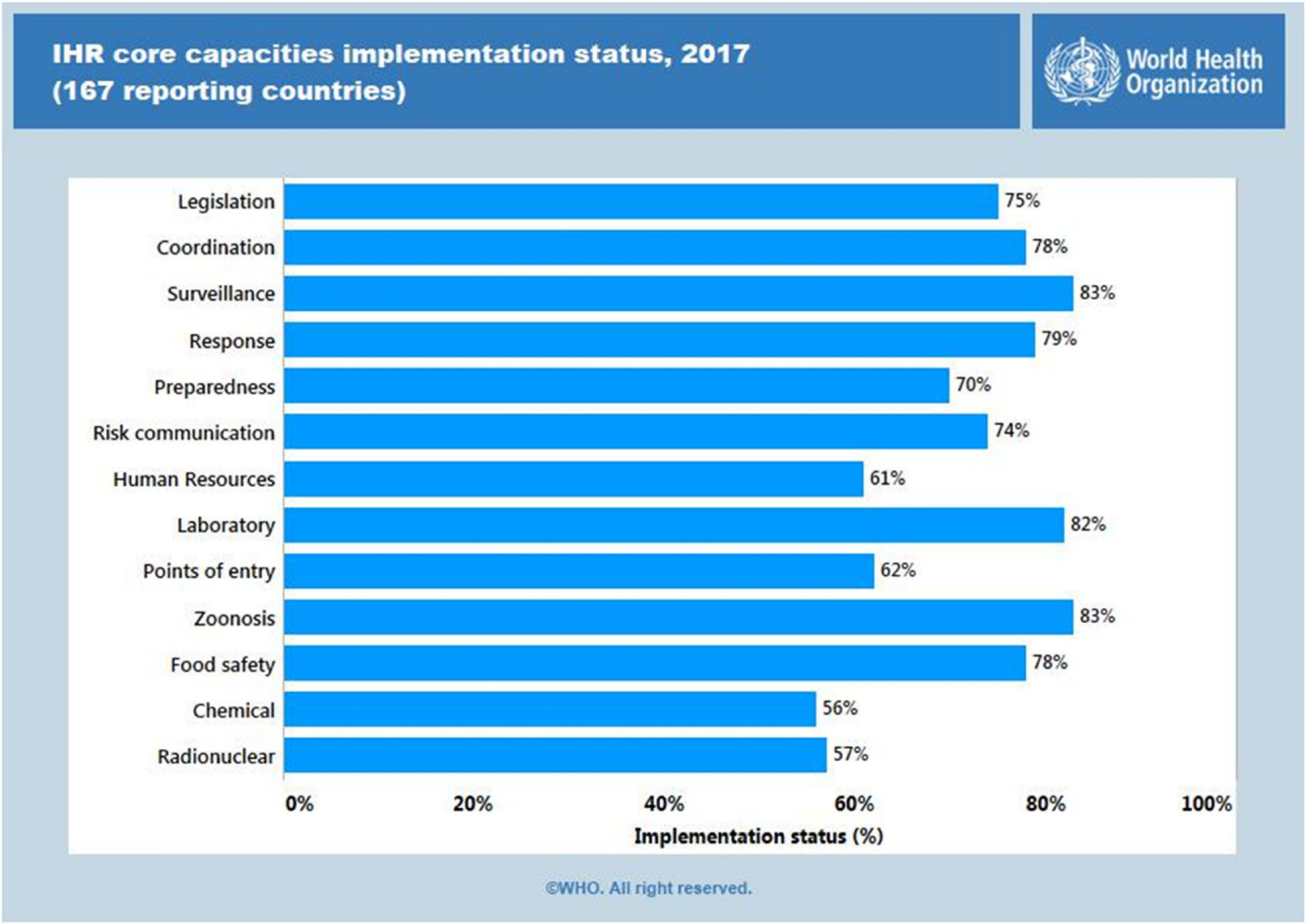
The implementation and monitoring of core capacities continues to present a challenge in many technical areas (Source: The World Health Organization)

The implementation and monitoring of core capacities continues to present a challenge in many technical areas, including adoption of national legislation, ensuring effective surveillance and response, establishing laboratory capacity, human resource development and ensuring biological, chemical and radio-nuclear safety (Fig. 6):

Effective multisectoral collaboration remains a priority. WHO, its partners and Member States must continue working collectively to bridge identified gaps in IHR core capacities in the most efficient and effective way, using existing strategic approaches, networks and resources. Greater international cooperation and coordination, additional tools, further research are required. This can be partly achieved within a health security interface conceptual framework.

#### WHO health security interface

WHO’s Health Security Interface (HSI) Secretariat core strategic activities can be separated into three main areas of work and are pursued simultaneously: Advocating for the inclusion of public health in the security sector as a way to reduce global security risks; Increasing WHO preparedness and response measures to public health emergencies including deliberate events; and providing outreach activities within the organization to raise the awareness and visibility of the health security interface as a vital component of WHO.

In practice this means improving coordination of HSI-related activities through the HSI Secretariat and increasing WHO’s global footprint on HSI preparedness at the policy level. Another key area is to develop partnerships with entities providing support in infectious disease outbreaks, especially those of a deliberate nature, including in the context of mass gatherings. Monitoring innovations in science and technology is also a concrete action, which includes providing timely analysis of health security interface implications.

HSI has provided ongoing advice to WHO/WHE staff regarding arising events and implementation of WHO Resolution WHA 55.16 which relates to global public health response to natural occurrence, accidental release or deliberate use of biological and chemical agents or radio-nuclear material that affect health.

#### HSI activities include

- Outbreak response operations in non-permissive environments, such as highly politicized contexts, conflicts, and wars
- Deliberate events, including the intentional use of chemical or biological agents in order to cause harm
- Issues related to mass gatherings, such as major sporting events, festivals, and regular religious migrations
- Supporting Member States through a Self-Assessment Tool, modelled on the JEE, to assess its specific preparedness and response capabilities for deliberate events.
- Awareness Raising and Training for WHO staff and Member States on all aspects of health security issues, including deliberate events.

WHO HSI engages in multi-sectoral collaboration with organizations such as Organisation for the Prohibition of Chemical Weapons (OPCW), United Nations Office for Disarmament Affairs (UNODA), United Nations Office of Counter-Terrorism (UNOCT), The Biological Weapons Convention Implementation Support Unit, United Nations Office for the Coordination of Humanitarian Affairs (UNOCHA), Global Outbreak Alert and Response Network (GOARN), Food and Agriculture Organization (FAO), Organization of Animal Health (OIE), International Criminal Police Organization (INTERPOL), the International Committee of the Red Cross (ICRC) and others. Informal contact between these organizations enables the sharing of information on events of potential deliberate nature.

HSI has issued an internal report entitled *Does (Public) Health and Security Interface Matter?,* which led to an accepted definition of the Health Security Interface within WHO. The unit has established an operational health/security secretariat allowing the future coordination of activities related to deliberate events across headquarters, regional and country offices, as well as the external coordination with outside organizations.

In late 2018, WHO has established the HSI Technical Advisory Group (HSI-TAG) to provide advice to WHO and the WHO Health Emergencies Programme (WHE) across areas related to the interface between the public health and security sectors. The group will act as an advisory body to WHO to strengthen internal capacities in the health security related fields, including deliberate events. It will also provide recommendations, as necessary, on matters related to tools, resources and systems available to WHO. The 17 members of the HSI-TAG are all senior subject matter experts (SMEs)_representing a broad range of different scientific areas, public health and the security sector.

The future of biological threat reduction requires sustained multisectoral cooperation and commitment at the national, regional and global levels.

### HIGHLIGHT: strengthening global biological security

*Trevor Smith, Global Affairs Canada*

The threats posed by biological weapons are very real; collaboration at the global health-security interface can play a key role in mitigating all manner of biological threats, whether natural, accidental or deliberate in origin.

Since the dawn of time, disease has shown itself to be an extremely efficient and effective weapon of mass destruction. Diseases, particularly those caused by high consequence pathogens, are relentless and adaptive and have no regard for geographic boundaries or political affiliations. For these reason, biological agents have consistently attracted the attention of states, terrorist groups and individuals with an interest and intent to cause harm, panic and/or disruption on a wide scale.

### The threat

During the twentieth century, numerous countries pursued biological weapons (BW) programs, seeking to harness the destructive power of disease for military purposes. In an effort to halt this dangerous trend, the Biological and Toxin Weapons Convention (BTWC) was negotiated and entered into force in 1972, with the aim of prohibiting the development, production, acquisition, stockpiling and retention of biological weapons. While the BTWC currently has 183 States Parties, it lacks a mechanism by which to independently verify compliance, and multilateral efforts to meaningfully strengthen the Convention have made little progress in the past two decades.

The BTWC was intended to prevent States from developing biological weapons, but non-state actors have also demonstrated the desire to acquire and the willingness to use biological weapons. In September 2001, letters containing anthrax were mailed in the United States, killing five people and infecting 17 others. Though only 5 g of infectious material was used, the impact of the “Amerithrax” letters went far beyond the immediate victims: tens of thousands of people received antibiotics, millions were terrified and billions of dollars were spent to deal with the immediate and longer-term consequences (including hundreds of millions of dollars to decontaminate affected post offices). In this regard, the anthrax letters demonstrated that even a modest scale deliberate biological event can cause mass disruption and huge economic and social impacts. Not surprisingly, the threat of deliberate use of disease as a weapon remains an attractive option for many terrorists, as evidenced by the numerous thwarted incidents in recent years.

#### Natural “proliferation events”

The challenges posed by biological weapons proliferation and bioterrorism are daunting, particularly given the natural origin and widespread availability of pathogens, which are the key ingredient required for development of a bioweapon. From this perspective, naturally occurring outbreaks of especially dangerous pathogens must be viewed as potential “proliferation events”, as they generate samples that could be used to develop a capacity for biological weapons or bioterrorist attacks. The scale of the problem is evidenced by the 2014–2016 Ebola outbreak in West Africa, which produced an estimated 300,000 Ebola samples, stored in dozens of facilities across multiple countries. In West Arica, as in many other parts of the world, facilities housing Ebola and samples of other pathogens of security concern often have inadequate or no biosafety, biosecurity or tracking measures in place to ensure that these “dual use” materials remain out of the hands of those with malign interests.

#### A global partnership against weapons of mass destruction

In the wake of the terrorist attacks of September 2001 in the United States and the subsequent “Amerithrax” letters, the then G8 group of nations came together to take meaningful action to combat the threat of terrorist use of chemical, biological, radiological and nuclear (CBRN) weapons. At their 2002 Summit in Kananaskis, Canada, G8 Leaders launched the *Global Partnership Against the Spread of Weapons and Materials of Mass Destruction* (GP) with a mandate to prevent terrorists and those that harbor them from acquiring weapons and materials of mass destruction and their means of delivery. Initially created as a 10-year, $20 billion initiative with a focus on CBRN-related challenges in Russia and other countries of the former Soviet Union, the Global Partnership delivered impressive results that eliminated or mitigated a wide range of serious WMD threats. As a result of its success, the GP was extended and expanded to address threats posed by weapons and materials of mass destruction on a global basis. While the Global Partnership remains a G7-led initiative, it now includes 30 active member countries plus the European Union. To date, GP members have delivered more than $25 billion in concrete, tangible programming worldwide in efforts to prevent chemical, biological, radiological and nuclear proliferation and terrorism.

In 2010, Global Partnership member countries agreed to place increased emphasis on biological threat reduction, and identified “strengthening biological security” as a key priority for collective programming efforts. The GP developed a set of five biological security “Deliverables” (which were reviewed and updated during Canada’s G7 Presidency in 2018) that commit GP partners to support efforts to:

- Secure and account for materials that represent biological proliferation risks;
- Develop and maintain appropriate and effective measures to prevent, prepare for, detect and disrupt the deliberate misuse of biological agents;
- Strengthen national and international capabilities to rapidly identify, confirm/assess and respond to biological attacks;
- Reinforce and strengthen the BTWC and other biological disarmament and non-proliferation obligations, principles, practices and instruments; and
- Reduce biological proliferation risks through the advancement and promotion of safe and responsible conduct.

A core principle of GP efforts to mitigate biological threats and to strengthen global biological security is the importance of robust partnerships with the health sector (i.e. human, animal and plant). In this regard, GP partners have forged enduing, mutually-supportive collaborations at the “health-security interface” at the global (e.g. WHO, OIE and FAO), regional (e.g. Africa, Asia, Caribbean) and national levels. GP partners recognize the imperative for stakeholders from diverse sectors and backgrounds (e.g. human health, animal health, academia, development, defense and security) to work together to prevent, detect and respond to all manner of biological incidents (whether natural, accidental or deliberate in origin) and to build impactful and sustainable health-security capacity.

#### The role of Canada

Canada’s Weapons Threat Reduction Program (WTRP), originally known as the Global Partnership Program (GPP), is Canada’s contributions to the G7-led *Global Partnership Against the Spread of Weapons and Materials of Mass Destruction*. Since its establishment in 2002, Canada’s WTRP has delivered nearly $1.4 billion in concrete programming to address chemical, biological, radiological and nuclear proliferation and terrorism threats. The WTRP was renewed on an ongoing basis in 2018 with funding of $73.4 million per year.

Canada’s WTRP is a leader in the delivery of biological threat reduction programming and has supported dozens of countries over the years to, inter alia, strengthen biosafety and biosecurity for pathogens of security concern (e.g. anthrax and Ebola), enhance surveillance and diagnostic capabilities and improve capacities to prevent, detect and respond to biological threats. Major initiatives currently underway include projects to identify, secure and/or destroy vulnerable samples of Ebola virus in Africa support to the WHO and other international agencies to develop a new capacity to respond to the deliberate use of disease and the development of new solutions to enhance sustainable biosafety and biosecurity in low resource settings. In the delivery of global health security programming, Canada’s WTRP has developed strong ties and collaborative partnerships with other Government of Canada agencies (e.g. Canadian Food Inspection Agency, Public Health Agency of Canada and the National Microbiology Laboratory in Winnipeg), international organisations (e.g. WHO, OIE and INTERPOL) and other GP partner countries (most notably the United Kingdom and the United States).

#### From slogan to reality

In recent years, considerable progress has been made in acknowledging the importance of multisectoral collaboration and partnership to mitigate all manner of biological threats, regardless their origin. However, while initiatives such as the Global Health Security Agenda (GHSA) have elevated the level of attention and political discourse, more needs to be done to translate high-level commitments into tangible actions. Biological threats persist and are ever-evolving, and no single sector, country, agency or organization can hope to mitigate them alone. Further work is required to create a true global health security partnership, where stakeholders from across the spectrum come together for a common objective: enhanced capacity and capability to prevent, detect and respond to all manner of disease. Only through more active, coordinated and mutually beneficial collaboration will “global health security” be converted from a slogan to an actuality.

### ACTION: installation of a bio threats scanning group

The **Bio Threats Scanning Group** comprises top level experts from 10 leading laboratories: Linfa Wang (Duke-NUS Medical School, Singapore); George Gao (China CDC); William B. Karesh (EcoHealth Alliance); Ab Osterhaus (RIZ Hannover, Germany); John Mackenzie (Curtin University, Australia); Rebecca Katz (Georgetown University, US); Ron Fouchier (Erasmus MC Rotterdam, Netherlands); Thomas V. Inglesby (John Hopkins, US); Geoffrey Smith (Cambridge University, UK); Ian Lipkin (Columbia University, US). It was established

- to provide an overarching perspective to One Health and global health security,
- to connect One Health science and Global Health Security policy,
- to help predict potential biological events that could impact on public health security in the future
- to actively collaborate on bio threats scanning with international organizations.
- to take up an Ad Hoc advisory role provided on an on-call basis at external meetings or to groups (both scientific and policy)
- to safeguard consistent attention for global health security throughout the congress programmes

### ACTION: connecting one health science and global health security

There is a significant increase in the emergence of infectious agents and the risk of new pandemics as exemplified by the spread of highly pathogenic H5N1 influenza since 2003, the pandemic H1N1 influenza in 2009, influenza H7N9 in 2013, SARS, MERS, chikungunya, dengue, Zika and Ebola. It is relevant to note that SARS, as the first novel pandemic virus of the new millennium, has clearly demonstrated that:

- previously unknown pathogens can emerge from a wildlife source at any time in any place and without warning, threaten the health, well-being and economies of all societies;
- there is a clear need for countries to have the capability and capacity to maintain an effective alert and response system to detect and quickly react to outbreaks of international concern, and to share information about such outbreaks rapidly and transparently;
- responding to pandemic threats requires global cooperation and global participation.

Zoonotic diseases have security implications: 80% of known biological weapons have a zoonotic origin. Yet, the One Health concept is not limited to zoonoses as it indeed incorporates all pathogens which have an impact on Global Health Security, including food and water security.

The essential need for a multilateral and multi-sectoral approach to prevent, detect, and respond to infectious diseases threats is acknowledged by all major international health organizations, including the World Health Organization, the Food and Agriculture Organization, the World Organization for Animal Health, the World Bank, the Biological and Toxin Weapons Convention (BTWC) and the Global Health Security Agenda (GHSA). It is also at the core of the Global Partnership Biological Security Working Group objectives, as it aims to build global capacity to prevent, detect, and respond to deliberate disease threats.

The One Health Platform is a Scientific Reference Network and a Strategic Forum of Stakeholders that connects One Health Science and One Health Policy to improve Global Health Security.

In the 2019–2020 period, the One Health Platform aims to:

- Exchange the latest high-level scientific state-of-the-art research and gather information on OHS and AMR at the biennial congresses and disseminate the results and insights of existing and new research projects on zoonoses, emerging infectious diseases and antimicrobial resistance, including the ecological and environmental factors which drive and impact on these diseases. In that way, we aim to make science evolve, thus improving health and security;
- Identify and prioritize research gaps in the fields of zoonoses, emerging infectious diseases and antimicrobial resistance, including the ecological and environmental factors which impact on these diseases, and advocate the resulting scientific research agenda - both at the scientific and the policy level;
- Engage the broader scientific community and global health security policy makers in understanding that some zoonotic and animal diseases can potentially be misused;
- Work with the Bio Threats Scanning Group of experts further connecting One Health Science and Global Health Security Policy;
- Create synergies and facilitate the sharing of data between researchers and research groups in order to mend the prioritized research gaps, and translate these novel data to anyone who might benefit from them;
- Translate the relevant information and knowledge to governments and policy makers and serve as a reference network and a resource for providing information to governments, thus connecting One Health Science and Global Health Security Policy by maintaining a web portal and by operating a Customer Relationship Management (database) system;
- Provide a strategic forum for researchers, early career investigators, governmental and non-governmental institutions, international organizations and companies in the One Health arena to foster cross-sectoral collaborations.

## Reducing antimicrobial resistance

### HIGHLIGHT: quantifying the problem of antimicrobial resistance

*Joergen Schlundt, Nanyang Technological University, Singapore*

This highlight presents an overview of antimicrobial resistance, and a plea for its One Health surveillance and the use of next generation DNA sequencing as a tool to respond to it.

Antimicrobial resistance (AMR) is a fast-growing problem caused by a number of complex and interacting factors involving different sources and transmission pathways. The lack of data on the relative importance of these different pathways is hampering global efforts and optimal interventions to effectively manage the risks. In particular, little quantitative knowledge exists on the relative contributions of these sources, which means that targeting interventions could be sub-optimal.

#### The origins of AMR

Originally it was suggested that the AMR problem originated from the use of antimicrobials in humans, but human use is now known to account for only part of the problem.

**Fig. 7:**
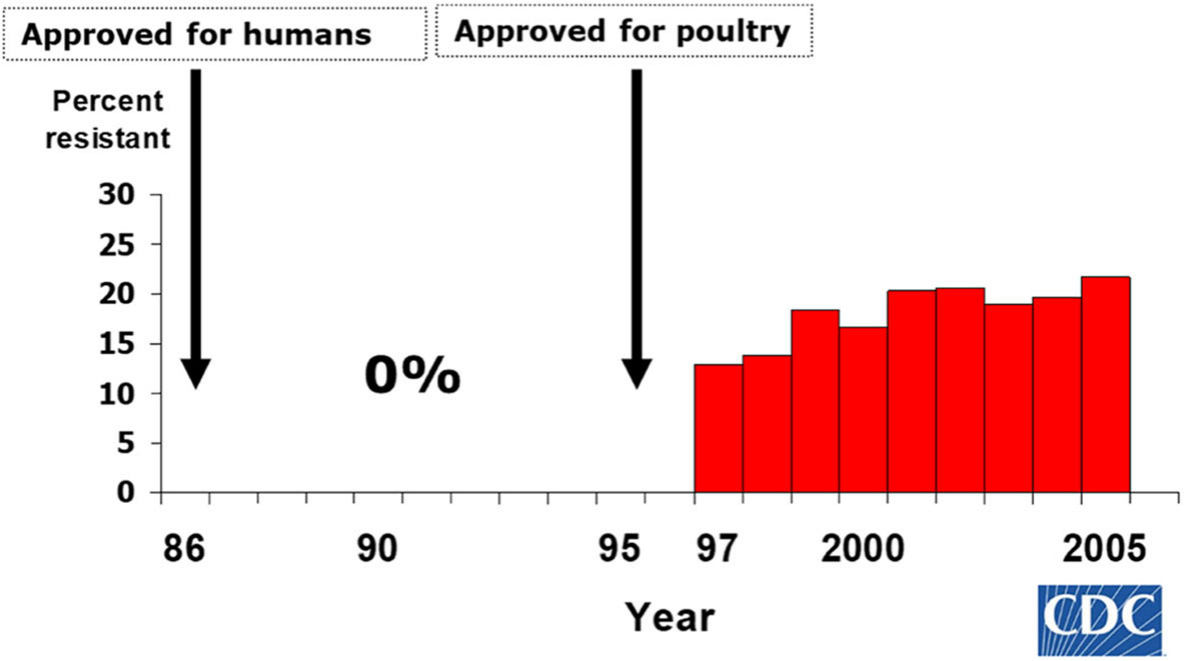
Percent fluoroquinolone resistant Infections In humans In USA 1986–2005 (Source: CDC)

Fluoroquinolone was approved for human use in the US in 1986 and over the next 10 years, no resistance in human infections was observed. In 1996 it was approved for use in poultry and resistant infections in humans were immediately noticed (Fig. 7).

It has also been documented how reduction in use results in reduction in AMR. The prevalence of nalidixic acid resistance in *Salmonella* isolates from chicken in China and Europe, where no restrictions on its use are in force, is respectively 67 and 62%. In the US where its use in poultry is banned, resistance is only 1.5%. It should be noted that basically all human salmonellosis cases originate in food-producing animals [11].

**Fig. 8:**
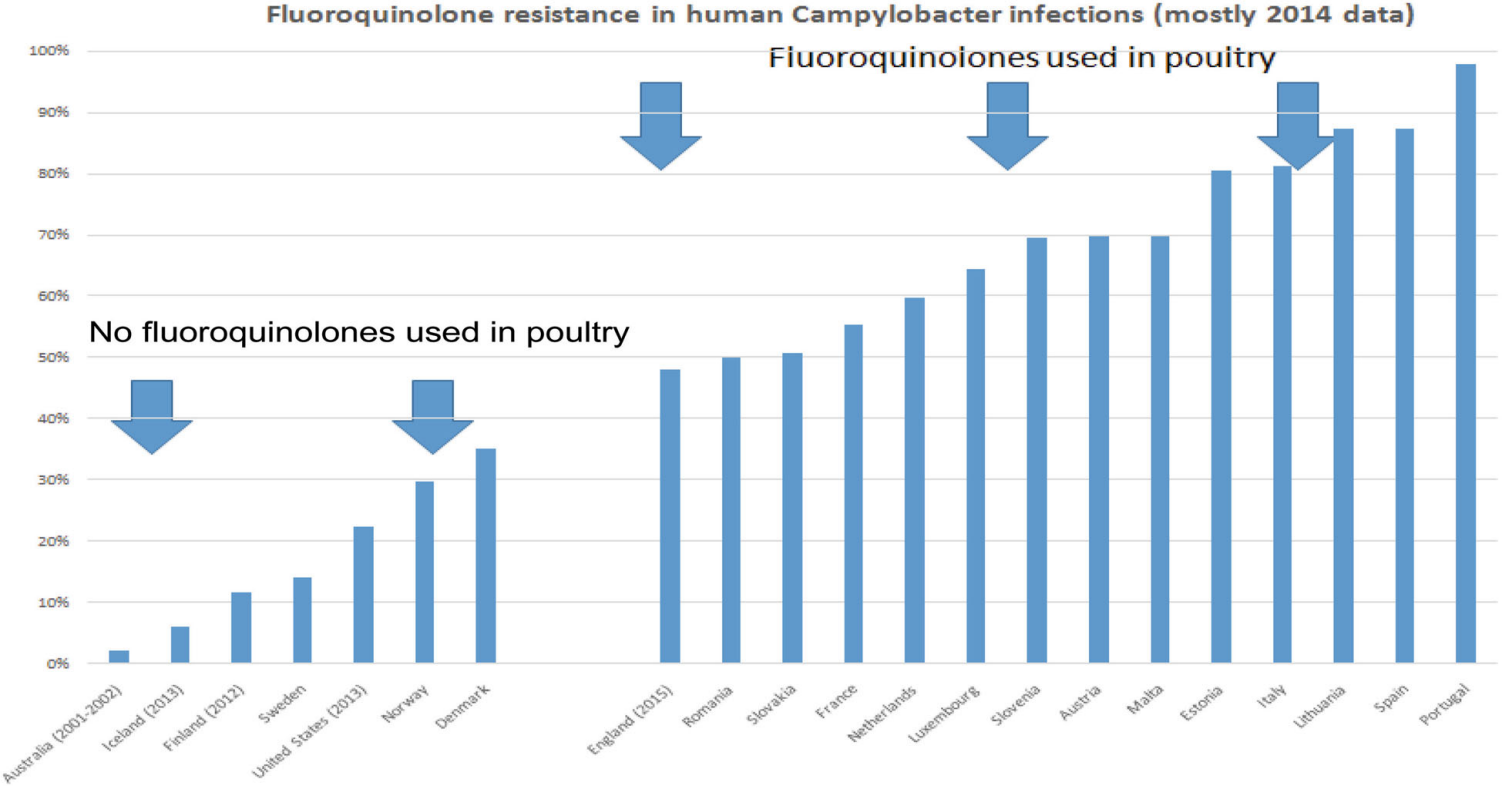
Fluoroquinolone resistance in human Campylobacter jejuni infections (ECDC 2014 https://www.ecdc.europa.eu/en/antimicrobial-consumption/surveillance-and-disease-data/database)

Evidence for resistance transmission from farm animals to humans is particularly strong in the case of *Campylobacter* relative to the use of antimicrobials in poultry. The difference in resistance to fluoroquinolones in human *Campylobacter* infections in countries that have banned or still allow their use is clearly evident (Fig. 8).

Despite the evidence, discussions are still ongoing. For example, industry advocates claim that the increase in fluoroquinolone resistance in human *Campylobacter* infections in Denmark in recent years (to 35%), despite the fact that fluoroquinolones are not used in Danish poultry, indicates that it is human fluoroquinolone use which is to blame. However, research data shows that the most likely explanation for the trend is the large increase in poultry imports to Denmark from countries that use fluoroquinolones in poultry.

Similarly, production of extended-spectrum β-lactamases (ESBLs) is a significant resistance mechanism that impedes the antimicrobial treatment of infections caused by *Enterobacteriaceae* by cephalosporins. Neither Denmark nor Sweden use cephalosporins, yet resistant microorganisms in live poultry moving from the UK (where cephalosporins are used) to Denmark and Sweden have been identified, resulting in the introduction of such resistant microorganisms in production chains in Denmark and Sweden. This indicates that a problem with AMR in one country is a problem for all countries, especially with global exports of food and animals rising.

#### The impacts of AMR

Widely ranging estimates exist for the importance of the food animal reservoir of resistant microorganisms for human health. Industry organizations claim almost zero deaths, whereas specific research focusing on cephalosporin resistance alone indicates that more than 1500 deaths and over 65,000 days of hospital admissions are due to the use of cephalosporins in chickens in Europe alone. The global estimate of deaths from AMR Is presently 700,000, suggested to rise to 10 million by 2015 [12] (higher than the 7–8 million cancer deaths).

Back in 2003, the 1st Joint FAO/OIE/WHO Expert Workshop on non-human antimicrobial usage and resistance concluded that there is clear evidence of adverse human health consequences caused by resistant organisms resulting from non-human usage of antimicrobials. This includes infections that would not have otherwise occurred; increased frequency of treatment failures and deaths; and increased severity of infections (e.g. fluoroquinolone resistant *Salmonella* infections).

A Danish study concluded that patients infected with *Salmonella typhimurium* resistant to ampicillin, chloramphenicol, streptomycin, sulfonamide and tetracycline were approximately five times more likely to die than the general population within the 2 years following infection, whereas patients infected with quinolone resistant *S. typhimurium* were approximately ten times more likely to die than the general population – patients infected with fully susceptible *S. typhimurium* were only two times more likely to die within 2 years [13].

A comparison of two hospital cohorts (combined from 13 countries) showed that mortality associated with *E. coli* infections resistant to 3rd generation cephalosporins was 2.5 times higher than for patients infected with susceptible *E. coli* [14].

Moreover, it is estimated that at least 23,000 deaths per year in the US and up to 25,000 deaths per year in Europe could be directly attributable to AMR. Obviously, this has a huge economic impact too. The costs attributed to resistant bacterial infections in humans were in 2009 estimated at €1.5 billion annually in Europe [15], and $20 billion annually (for healthcare) and $35 billion a year (productivity loss) in the USA [16]. Some models estimate that AMR has a cost of more than 0.5% of the global GDP [17]; a figure that is likely to increase significantly in the coming decades.

#### Steps to tackle AMR

The Global Action Plan to tackle AMR was endorsed by the World Health Assembly in May 2015. Its five objectives are to improve awareness and understanding of AMR; strengthen knowledge through surveillance and research; reduce the incidence of infection; optimize the use of antimicrobial agents; and ensure sustainable investment in countering antimicrobial resistance.

In the absence of a good evidence base it is very difficult to compare the effectiveness of different initiatives in different countries [18]. However, important steps already taken are:

- documenting the use of antimicrobials and the occurrence of resistance;
- regulating all use of antimicrobials;
- blocking the rights of vets and physicians to make a profit from selling antimicrobials;
- significantly reducing the unnecessary use of antimicrobials (starting with a ban on the use of antimicrobial growth promoters).

In regard to stopping the veterinary profession from profiting from selling antimicrobials, in 1995 a law was passed in Denmark that banned the routine prophylactic usage of antimicrobials in animals, and which limited the veterinarians’ profit from the direct sale of drugs. This immediately led to a 40% drop in therapeutic drug use in the animal sector in Denmark. Similar bans exist in other Nordic countries. Most likely strong opposition from the veterinary organizations – and drug companies – have resulted in that such restrictions have not been accepted in most other countries.

#### Global microbial identifier (GMI) (www.globalmicrobialidentifier.org)

**Fig. 9:**
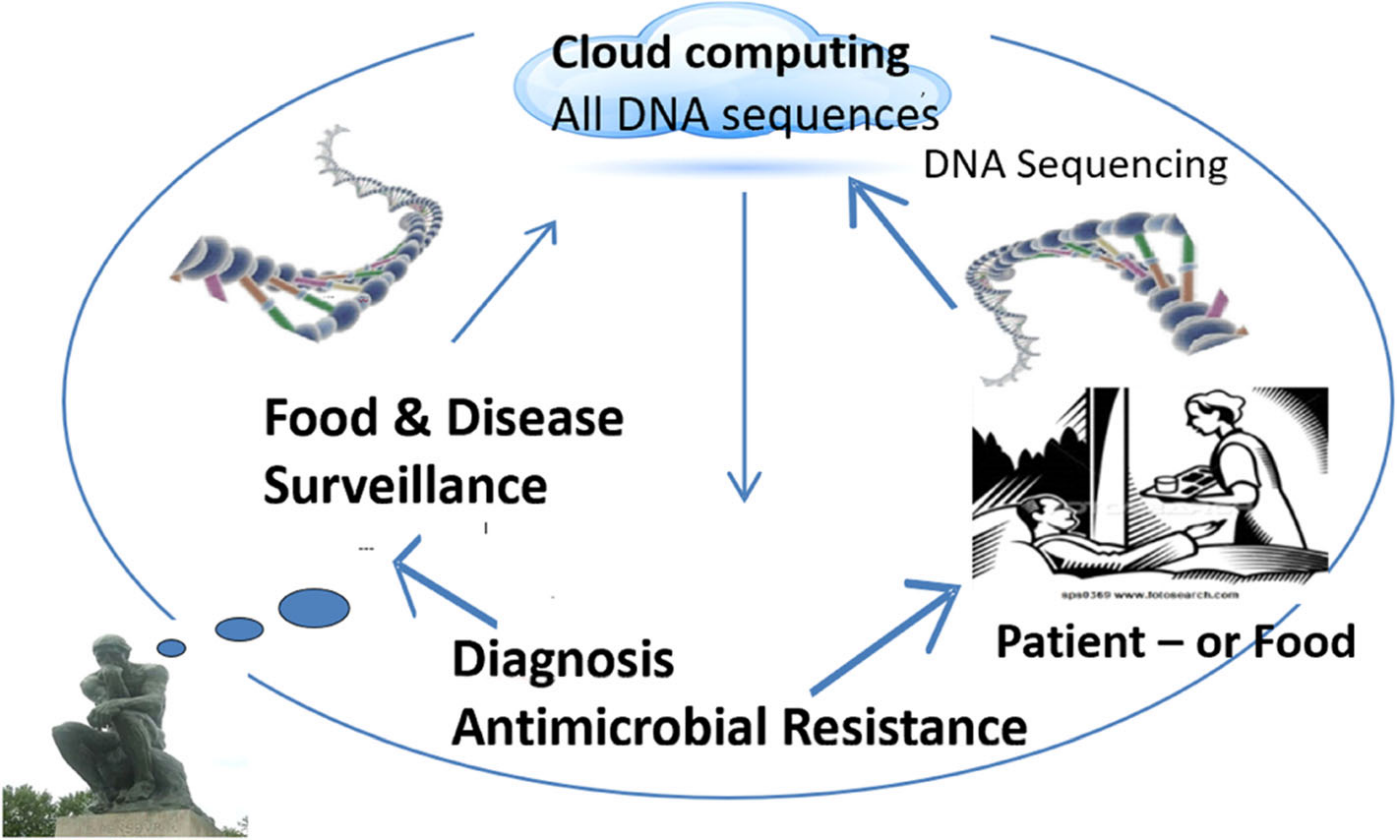
In the future, a global system of DNA genome databases for microbial (and AMR) identification and diagnostics could be established, enabling a professional response to health threats for all countries with basic laboratory infrastructure

In the future, a global system of DNA genome databases for microbial (and AMR) identification and diagnostics could be established, enabling a professional response to health threats for all countries with basic laboratory infrastructure (Fig. 9).

A GMI would enable five major lines of action:

- simple identification of all microorganisms in clinical (or other) settings;
- enabling reduction of total time (and cost) for characterization;
- a total database of unique sequences of all relevant microbiological strains globally – including ‘positive’ microorganisms, e.g. for food production or environmental remediation;
- enabling *real-time* global surveillance of human, animal and plant disease developments;
- and a backbone of microbial DNA sequences to be used for deep sequencing analyses from different sources (gut, sewage, environment, food) [19].

Developing countries could enjoy a next generation sequencing leap-frog: new diagnostics systems avoid the need for specialized training and reach further with real-time characterization of microorganisms in decentralized labs with sequencers.

A global initiative to create an active database such as this will promote One Health as it can be applied to humans, animals, plants, food and the environment as well as to viruses, bacteria, fungi and parasites.

### HIGHLIGHT: the role of diagnostics and vaccines in reducing antimicrobial use in food producing animals

*Jaap A. Wagenaar, Utrecht University, the Netherlands*

Vaccination as a measure in infection prevention is generally considered to contribute to a reduction of antimicrobial use (AMU). One of the objectives of the World Health Organization Global Action Plan is to “reduce the incidence of infection through effective sanitation, hygiene and infection prevention measures”. In this plan, vaccination is mentioned as a tool to reduce AMU in humans as well as in animals (WHO-Global Action Plan). Vaccination against pathogens is a significant part of farm animal management. It should be stressed that vaccination is not a stand-alone measure but should be part of an integral approach including veterinary oversight, good biosecurity and husbandry practices, feed quality, climate and the use of diagnostics (OIE, ad hoc group, 2015). However, no quantitative data exists on the contribution of vaccination to the reduction of AMU.

Studies on the association between AMU and vaccination are scarce. One study explored whether the use of group medication with antibiotics in a Danish pig herd would be reduced after vaccination of the pigs against proliferative enteropathy (PE) caused by *Lawsonia intracellularis* [20]. In the vaccinated batches, the consumption of oxytetracycline to treat PE was reduced by 79%, with a significantly lower number of pigs being treated (*P* < 0.0001). Vaccination thus resulted in a highly significant reduction in antimicrobial use.

One Danish study showed a positive association between the use of vaccines against Circovirus and *Mycoplasma* and the amount of antimicrobials prescribed in weaning pigs.

The same group retrieved data from Vetstat and studied the change in amounts of antimicrobials prescribed for weaners and finishers in herds following initiation of vaccination against five common endemic infections: *Mycoplasma hyopneumoniae, Actinobacillus pleuropneumoniae*, porcine circovirus type II, porcine reproductive and respiratory syndrome virus, and *Lawsonia intracellularis* [21]. This study provided little support for the hypothesis that vaccination against five common endemic diseases provides a plausible general strategy to reduce antimicrobial use in Danish pig herds.

A cross-sectional study in 227 farrow-to-finish pig herds in Belgium, France, Germany and Sweden between December 2012 and December 2013 [22] showed that herds vaccinated against more pathogens showed a higher treatment incidence.

Summing up the scientific evidence, there seems to be a correlation between the use of vaccines and the higher use of antimicrobials. This is most probably the result of a response to disease problems.

#### Field observations

**Fig. 10:**
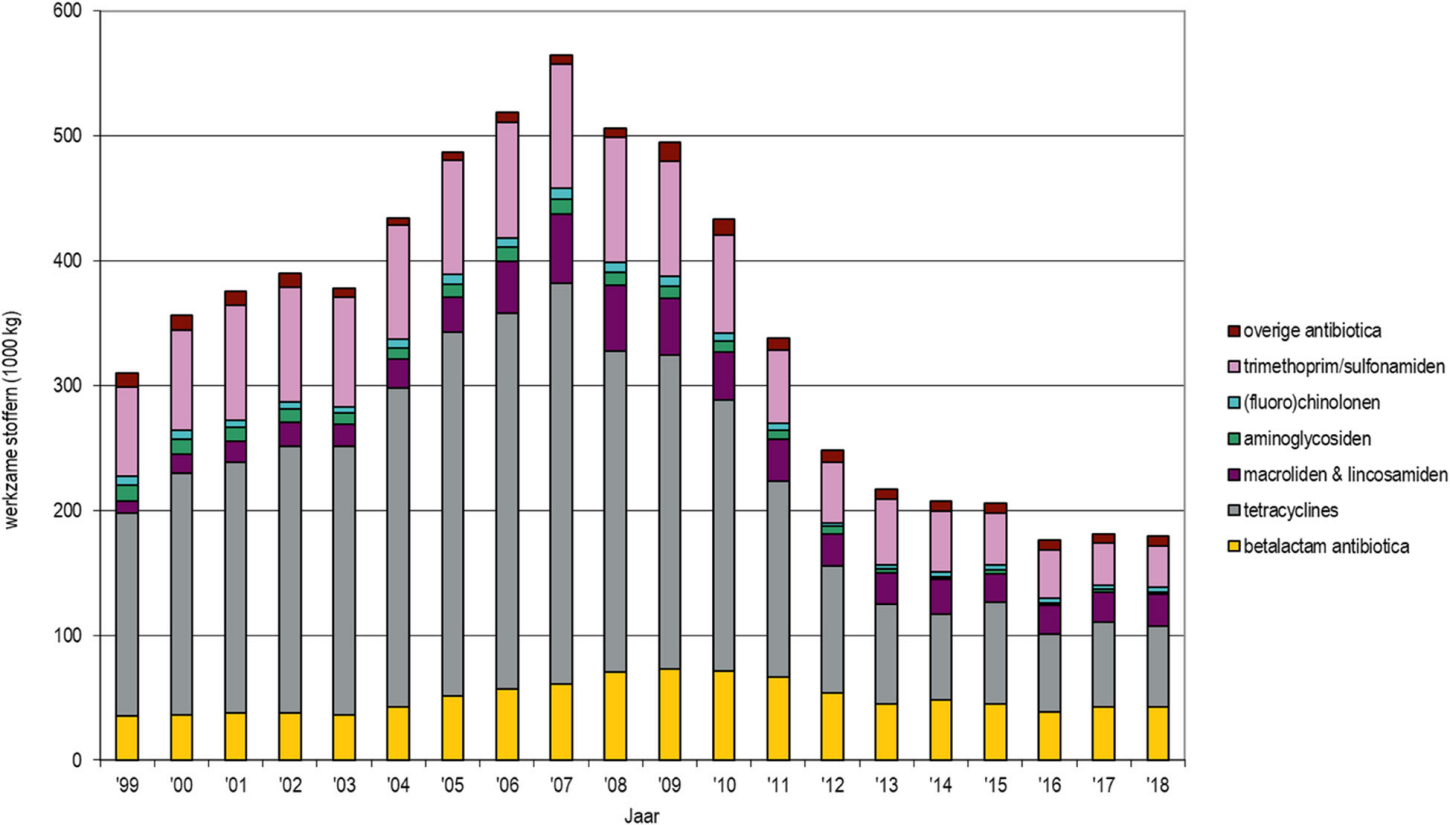
Total sales of antimicrobials for use in animals in the Netherlands (source: FIDIN)

A country-wide intervention study was conducted in AMU in the Netherlands. Total sales in therapeutically used antimicrobials decreased by nearly 64% over the years 2009–2017 (Fig. 10).

**Fig. 11:**
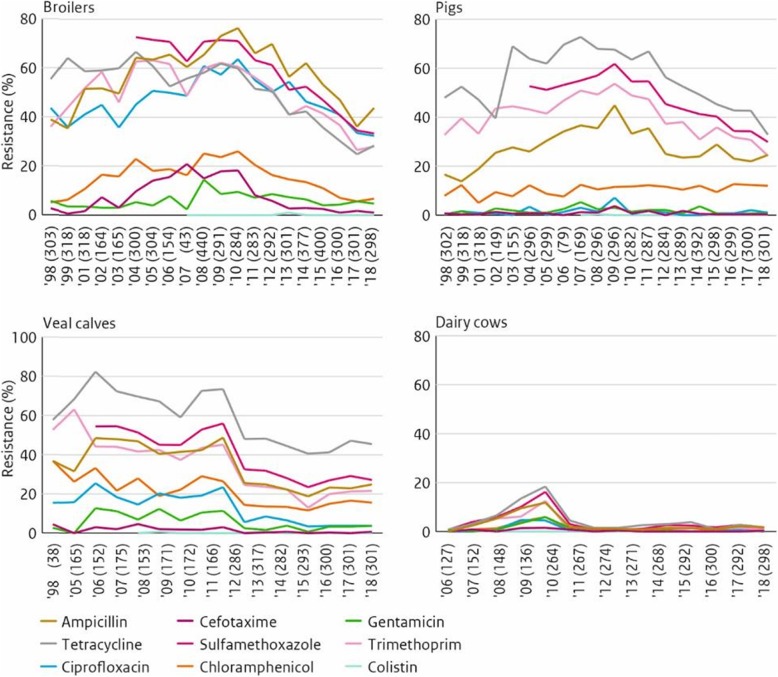
Antimicrobials resistance in commensal *E. coli* in animals (rectal swabs in slaughterhouse). Percentage of resistance for different antimicrobials in different animals

The decline in sales of antimicrobials was followed by a significant reduction in antimicrobial resistance as measured in commensal *Escherichia coli* in animals in slaughterhouse (NRL antimicrobial resistance, Lelystad, the Netherlands) [23] (Fig. 11).

Parallel to the decrease of AMU, data of vaccine sales from FIDIN, the federation of the Dutch veterinary pharmaceutical industry (2009–2017) showed an increasing use of vaccines in pigs (+ 100%), poultry (+ 25%), cattle (+ 100%) and goats (+ 6%).

It is interesting to note that, when farmers and veterinarians are asked about measures taken to reduce the use of antimicrobials, vaccination is often not considered to be an important measure. In broiler production the shift to slower growing breeds has led to a huge reduction in the use of antimicrobials in broilers. In the dairy industry, the introduction of a guideline for the selective drying of dairy cows instead of blanket therapy resulted in a considerable reduction in the use of antimicrobials. The reduction in AMU was 39% for dry cow therapy and of 38% for clinical mastitis cases.

#### Diagnostics

Diagnostics are an important tool in antimicrobial stewardship. Bacteriological analysis including antimicrobial susceptibility testing provides guidance for the choice of antimicrobial that can be used. In both the Netherlands and Denmark, a system is in place in which some antimicrobials are restricted to human use and only used in animals in exceptional cases. These are defined by the WHO as the highest prioritized Critically Important Antimicrobials for Human Medicine (CIA) and include 3rd and 4th generation cephalosporines and fluoroquinolones. These can only be used for animals when a susceptibility test has been conducted that demonstrates that there is no alternative first or second choice antimicrobial. This policy has led to hardly any 3rd and 4th generation cephalosporines and fluoroquinolones use in these countries.

#### Conclusions

Hardly any evidence-based data exists on AMU reduction by vaccination in animal production. Field studies are difficult to perform as they are influenced by so many factors. However, it is extremely important to stress that vaccination is just one element of an infection control program that should be guided and tailor-made by a professional (veterinarian). A professional who can oversee the different aspects is needed to give advice on the use of vaccines.

### HIGHLIGHT: reduced and responsible use of antibiotics in food-producing animals in The Netherlands

*Christianne J.M. Bruschke, Frouke de Groot, Ministry of Agriculture, Nature and Food Quality, The Netherlands*

This highlight provides an overview of past, present and future measures implemented in the Netherlands to reduce the usage of antibiotics and the levels of antimicrobial resistance.

The Netherlands is a densely populated country of 34,000 km^2^ and 17 million people. Next to the high number of people there are 4 million cattle, 12 million swine, 325,000 horses, 1.5 million sheep and goats, and 100 million poultry. Within a 10 km radius of some areas of the country up to 1000 commercial cattle and pig farms, or 400 poultry farms can be found. The high usage of antibiotics in the veterinary sector in the beginning of this century was related to a strong risk of emerging drug resistance. Consequently, there was a clear need for a reduced and responsible use of antibiotics in food-producing animals in the Netherlands. The main goal of the antibiotic reduction program was to prevent the development and spread of antimicrobial resistance and to increase overall animal health in the country.

Since 2009, the antibiotic reduction policy has been structured around a number of key elements. Self-regulation has been combined with public surveillance and enforcement. Transparency involves the mandatory registration of the use of all antibiotics in central databases. Benchmarking has been performed by the independent Veterinary Medicines Authority (SDa) of the Netherlands. Mandatory health and treatment plans have been instigated at farm level, involving one-to-one relationships between farmers and vets. Veterinary guidelines have been communicated, and private quality schemes developed. Reduction targets have been set, such as reducing antibiotic use by 20% by 2011; 50% by 2013; and 70% by 2015 compared to the reference amount of substances sold in 2009.

**Fig. 12:**
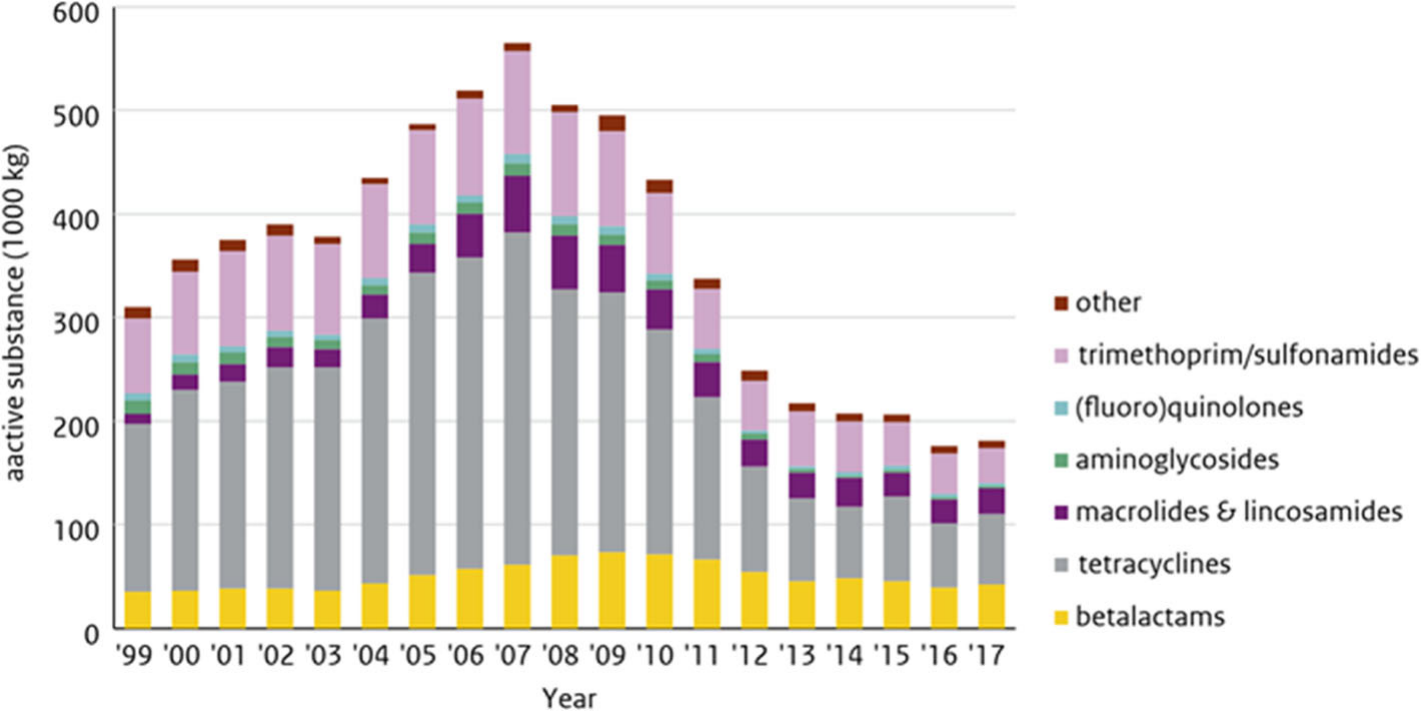
The Netherlands Veterinary Medicines Institute (SDa): Developments in sales of antibiotics between 1999 and 2018, in number of kilograms of active substances sold (× 1,000) (source: FIDIN), by main pharmacotherapeutic group

As a result, antimicrobial sales have significantly decreased over the last 10 years (Fig. 12).

By 2016 the use of antimicrobials had been reduced by 64%. In 2017 this level was slightly less, at 63.4%, The difference was explained by SDa as stockpiling and use in non-monitored sectors.

However, as can be seen from the graph, the reduction in the use of antibiotics levelled off in the past 5 years. The current policy was clearly no longer effective to further reduce antibiotic usage. As further reductions were still desirable to continue to reduce antimicrobial resistance, a new policy was necessary.

#### Key elements of reduction policy 2.0

Sector specific reduction actions along with research on the critical success factors that are correlated with the use of antibiotics will be used to further reduce the usage of antibiotics. However, a key question is “what are the critical success factors?”

Regarding the research being conducted into these critical success factors, it is useful to consider some of the methods employed. Data was collected on the use of antibiotics – whether low or high – on farms. Sectors covered were veal calf, pig and poultry. Statistical analyses were performed on possible associations between farm characteristics such as farm type, farm size, farm management, and animal health.

So-called ‘statistical associations’ refer to a certain factor related to the level of antibiotic use (this is not a cause-and-effect relationship). It may be that a ‘small size farm’ has an association with ‘low use’; this means that a smaller business in this sector may be a success factor to have. Note that the ‘why’ question has not been answered yet and must follow from further in-depth research, for example into behaviour.

The results of the critical success factors research are associations which are not necessarily causal relations. In all sectors, clear leads have been identified to decrease the usage. Further analysis on the associations is needed to define possible causalities. This has already started, focusing on social factors at farms with low usage. This will hopefully lead to the development of tools that could be implemented at farms with high usage.

Specific results from the veal calf sector indicate that small farms often have a lower usage of antibiotics, and that usage depends on management factors. Higher usage is associated with a lower number of staff; more calf nationalities present on the farm; calves from only Dutch origins; a higher number of bull calves; lower start weight; season (autumn and winter); and *Salmonella* infections. Also linked with management is that less antibiotics are used with a high number of Full Time Equivalents (FTEs) per calf, but other factors probably also play a role, such as education, personality type of farmer, etc.

Still in the veal calf sector, antibiotic usage also depends on social factors. Higher usage is associated with less trust in veterinarians; an increased role of a feeding specialist; a lower perception of the health of calves; and a decrease in social pressure. Other, unexplained factors affect higher usage, such as buildings staying empty for longer and physically being separated.

Looking at the pig sector, small farms often have a lower usage of antibiotics, and usage depends on a range of management factors. Fewer vaccinations give lower usage levels. For sows, higher usage occurs when fattening pigs stay on the farm. For fattening pigs, lower usage is seen when the animals are coming from closed farms. More piglets per sow increases the usage.

Turning to the poultry sector, it’s clear that large farms often have a lower usage of antimicrobials. Slower growing breeds use less antibiotics, while thinning the flocks seems to increase the usage.

#### Conclusions

The current reduction of antibiotic use remains at nearly 64%. This is still less than the target of 70% reduction from 2009 levels. Sector specific reduction targets should lead to this 70% reduction. Especially in the veal calf sector, useful outcomes have been identified to lower the usage. Implementation of critical success factors will help to reach the targets.

Further analysis on the associations is needed to define possible causalities. This has already started by focusing on social factors at farms with low usage. This will hopefully provide tools that can be implemented at farms with high usage.

### ACTION: establishment of a new scientific agenda on AMR at the 6th world one health congress

Antimicrobial resistance has emerged as a health issue in the last decades, but only in the last couple of years has there been an understanding that we are facing a post-antibiotic era, in which common infections and minor injuries, which have been treatable for decades, can once again kill.

Misuse and overuse of antibiotics in both humans and agriculture are the basis of the emergence of AMR. Whilst raising awareness of AMR is an important issue, new antibiotics need to be developed. World experts should elaborate on the use of antibiotics and the surge of AMR in food animals and humans and scan the horizon for new antibiotics and antivirals in a true One Health spirit.

The One Health Platform brings this issue to the forefront by installing a double AMR track at its World One Health Congress to increase public awareness for AMR (Table 1).

**Table 1:** Double AMR track proposed for the World One Health Congress in 2020

1.	Burden and Impact of AMR - AMR and global burden of disease - Global health security - Impact of AMR on animal production and food safety - Impact of AMR on global trade - Societal impacts from AMR - Economic impact
2.	Transmission human, animal, environment - What has been achieved, what is the evidence? - Systems epidemiology of transmission - Biosafety - Farm to fork to man and back? - Waste management and impact on AMR transmission
3.	Surveillance of AMR - Human (hospital, community), animal, environment - Use of whole genome sequencing for surveillance, one health microbiology, Global Microbial Identifier - Evolution of AMR - Gene mobilization factors (including waste management) - Integrated AMR and antimicrobial use surveillance - Big data
4.	Use of antibiotics - AMR and use of antibiotics for growth promotion - Role of antibiotics (including food safety) - Antimicrobial treatment of emerging MDROs - Antimicrobial stewardship - Infection prevention and control - Access to antimicrobial medicines - Quality of antibiotics
5.	Policy interventions - Global policy interventions, IACG - National Action Plans - National/regional experience in reducing antimicrobial use: Efficiency and Effect - AMR and SDGs - Regulatory interventions - Use of Big Data to guide interventions - Trade policy (impact on global trade) - Quality measures of antibiotics
6.	New economic models - Push/pull incentives for antibiotic drug and diagnostic development - Public private partnerships - Drug/Diagnostic partnerships - Economic impact assessment and modelling - Economic benefits - New models of trade
7.	Behaviour Change and Social Sciences - Antimicrobial stewardship - Public perception and education in all One Health sectors - Socioeconomic barriers and cultural differences - Consumer behaviour and pressure - Serious gaming - Design thinking
8.	Capacity building - Training the next generation - Building surveillance infrastructure - Implementation National Action Plans - One health approaches to teaching and training curricula
9.	Diagnostics and detection - Rapid, point of care, pen side diagnostics - Outbreak detection - Biomarkers of infection - Food safety and monitoring - Diagnostic stewardship
10.	Antibiotic drug development and manufacturing - Design and implementation of efficient clinical trials for novel antibiotics - Development of new antimicrobials - Addressing bacterial cell wall permeability - Manufacturing in LMICS - Waste/waste water treatment
11.	Alternative approaches to tackling resistant infections - Vaccines (against bacteria, viruses, etc) - Antibiotic alternatives (e.g. bacteriophages, immune modulators) - Non-traditional approaches for humans and animals - Genome editing - Animal models of disease

## Let’s talk science

*PETER DOHERTY, Professor of Microbiology and Immunology at the University of Melbourne* 2

### Communication

The One Health approach has always been a part of human civilisation. The rise of agriculture (about 10,000 years ago) saw the progressive move from the hunter/gatherer lifestyle to stable communities in villages and towns, while biblical dietary restrictions reflected an awareness of food-borne diseases. Modern experiments on bovines led to the vaccinations we have today. Addressing the connections between human and animal health and the environment, the One Health Approach recognises their interrelatedness and its inherent complexity. A multidisciplinary, collaborative and integrated approach to zoonoses research, policy and management needs to be developed.

Such an approach presents an unprecedented complex global challenge for collaboration among a wide range of stakeholders, for whom knowledge and experience is vital. The One Health Congress is an opportunity to promote a ‘paradigm of connectedness’ and bring stakeholders, scientists and interested parties together, but it is not the only way to reach them. Sustained and targeted communication of the science involved, by scientists for the public, is one of the most effective ways to spread the scientific and One Health messages. Only by doing so and educating people, can the fear and misinformation that often distorts scientific messages be addressed.

### The success of one health

Amongst other biomedical discoveries, advances in research in immunology, virology, epidemiology and related fields have enabled researchers to take great strides in fighting infectious diseases. By recognizing the possibilities inherent in interconnectedness of infectious diseases on the one hand and comparative medicine on the other, practices have been developed that have led to numerous medical breakthroughs. Scientists identified, for example, humoral immunity as the basis of vaccines and allowed us to eradicate two pathogens after world-wide vaccination programmes – smallpox in humans in 1980 and rinderpest in cattle in 2011. The smallpox vaccine was created with a production system that entailed scarification of the virus into calves and then administered to humans, a process similar to that employed by the first immunologists in the eighteenth century. The eradication of rinderpest was a much more targeted campaign using many more epidemiological principles, which resulted in a real triumph for international agencies and international cooperation.

Implicit in the One Health agenda is that challenges like pandemics are global and do not respect international boundaries. Inclusion of the One Health Principles within public health agencies as well as the continued cooperation between international agencies and governments have been very important in advancing the One Health project and have been key to its success.

A growing awareness of the interconnectedness between human health and the environment is also evident within the medical sciences and academia in general. The medical journal *Lancet* has instituted a 20-year programme where they will report every 2 years on the interface between climate change and human health. They will take a broad approach and include issues such as food and water availability, quality of water, social disruptions as well as focusing on the spread away from the equator of insect-born infections. By taking a One Health Approach, the journal provides a platform for scientists to engage with new fields and research.

One of the most successful ways that science has been communicated recently to the public has been through the online forum, ‘The Conversation’. This forum is a not-for-profit media outlet that uses content sourced from academics and researchers. Any person associated with an academic or research institution can write for the platform on any scientific, economic or cultural topic. A rigorous but amiable editorial process is followed in collaboration with experienced journalists, to ensure the production of quality articles accessible to the public. Furthermore, a conflict of interest statement is provided along with the name and association of the author, to ensure full transparency. All articles can be shared free of charge by other media outlets, provided that they do not modify the content. The ‘Conversation’ has a monthly online audience of 10.7 million users onsite globally, and a reach of 35 million people through republication.

### Challenges to one health

Communicating the values of vaccines, especially to people in advanced countries who have not seen the infections that plague many places in the developing world, is another challenge facing scientists and policy makers. Making scientific information available across traditional media such as newspapers remains a struggle and misinformation around scientific data persists. In fact, the problem is growing. There are regular measles outbreaks in Western countries as parents refuse to vaccinate their children. The problem is that some parents, holistic healers and even doctors and legislators, do not accept the value of vaccination. The information available about vaccination is often contradictory as some sources are neither legitimate nor transparent, leading to a suspicion and mistrust of the science and scientists.

The main pandemic threat to humanity is influenza, which has not sufficiently been acknowledged for the danger it poses despite the efforts of scientists. It is well-documented that new pathogens are emerging from nature constantly, but there is not yet consensus whether anti-viral small molecules (drugs) against all virus classes that are potential threats should be made and tested. What is agreed is that we need better and more rapidly produced vaccines, and a clearer understanding of pathogenesis for evidence-based therapy. This is also the case for HIV/AIDS and the hepatitis C virus, which have no vaccines yet, though there is a drug cure for HCV.

A threat to the creation of One Health policies and the global One Health agenda is a changing political landscape. Political movements that promote parochialism and xenophobia are on the rise and present a real obstacle in implementing international policies, while ignoring the scale of the problem. The mix of populism and neoliberal libertarian philosophies that are against all taxation and regulation could erode our capacity to respond effectively to global infectious disease challenges. Diminished funding for international agencies and monitoring systems, which compromise both major diagnostic laboratories and public health services, can only raise the global risk level.

Furthermore, increased limitation on research with dangerous pathogens, accompanied by fear mongering has significantly slowed research. There has been growing opposition to this within the scientific community, as the roll out of science-based strategies that could diminish threat levels has been delayed. It has also prevented research into GM technology that could correct micro-nutrient deficiency. This type of research holds numerous possibilities in terms of human health and well-being but is being thwarted by uninformed, or misinformed, attitudes.

Humanity is becoming much more conscious that it is on a warming planet and there are substantial changes in weather patterns. Droughts, as a consequence of climate change, are adversely affecting biomes. This increasingly contributes to the spread of infections, contrary to the assumption that insect-borne infections are correlated to high rainfall. In the case of the West Nile flavivirus outbreak in the US in 2012, a drought promoted the spread of infection as it brought wild birds (the hosts of the virus) to limited water sources where they spread disease to mosquitos, who in turn infected humans.

### Recommendations

The need for substantial change is obvious. The way humanity is generating and using energy should be reconsidered, as should the use of plastics, the question of waste disposal and of chemicals into the environment. Strategies to manage the global carbon challenge should be developed so that society can transition to an equitable green economy. These are issues that have to be addressed in a connected and in a global partnership way. To ensure long-term sustainable solutions to these challenges, political will and commitment is essential. This does not only mean developing and implementing the correct policies locally and internationally, but also ensuring that the resources are regulated and available for international bodies to continue their work.

A new social contract between science and society is the only way forward. To achieve this, communication of scientific discoveries and practices should improve drastically. Those working in the sciences need to think in terms of how they communicate what they do and why it is important. They should provide an understanding to people of what the issues actually are. The communication around vaccinations should also be intensified. They way to do so is not to speak about the vaccine itself, but to speak about what the virus actually does and how it does it. If the public understood the consequences of not being vaccinated, it would contribute to an informed dialogue about the potential benefits of science to human health.

To communicate their science efficiently and clearly, researchers should be trained in translating their findings into information that is easily accessible to the public. Science can be difficult and complicated for non-scientists, especially topics such as immunity and infections. One of the best ways to make scientific information available is by using visual representation or visual elements, like infographics, videos or illustrations. This is a medium that can be easily understood and disseminated, as has been proven by the success of ‘The Conversation’. Scientists that have the capacity themselves or have the resources to employ someone to translate the science into a visual language, should endeavour to share the information in the public sphere. The way people are accessing information is changing and scientists and science communicators should respond to that.

### ACTION: VAXVOX

**Fig. 13:**
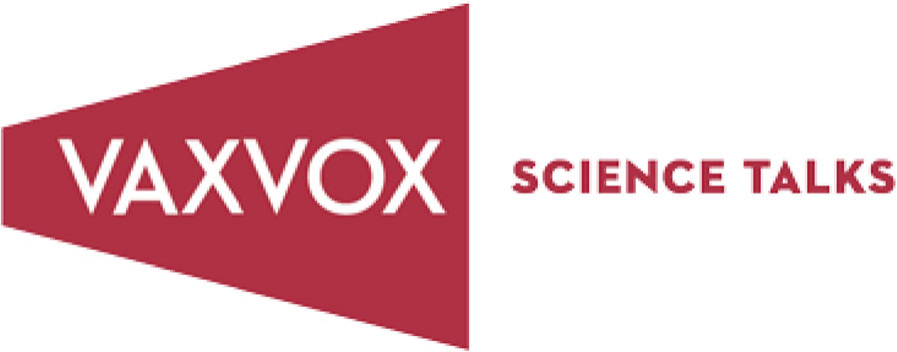
The logo of VAXVOX – Science Talks

To address the challenge of communicating the value of vaccinations in general, VAXVOX – science talks was installed within the One Health Platform (Fig. 13). The aim of VAXVOX will be to serve as a point of reference on the scientific aspects of the benefits of vaccination. VAXVOX will communicate pro-actively and will serve as a point of contact for the outside world on issues related to vaccination. In spreading scientific core messages,

Scientists have the moral obligation to “talk back”. Communication is crucial, sharing and explaining scientific insights are of critical importance. VAXVOX, thus meets the urgent need for a scientific organization that serves as a credible reference point on vaccines-related issues. By spreading scientific core messages, VAXVOX intends to raise a solid, united voice in the debate about vaccination.

## Conclusions: let’s continue the work

Many One Health issues and challenges were discussed during the 5th International One Health Conference in Canada. This document focused on some of the highlights. Again, it was clearly demonstrated that the majority of many new pathogens are zoonoses and an understanding of the interplay of factors at the interface between humans and animals is absolutely crucial for their detection, response and control. And this is exactly what One Health is about: an integrated, holistic approach. Indeed, the role of the wildlife-domestic animals-human-ecosystem interfaces has been fundamental to the development of the One Health paradigm.

Our important principles, taken from the Manhattan Principles on “One World, One Health” (2004) describe the fundamentals of One Health.

- To recognize the link between human, domestic animal, and wildlife health, and the threat disease poses to people, their food supplies and economies, and the biodiversity essential to maintaining the healthy environments and functioning ecosystems we all require
- To recognize that decisions regarding land and water use have real implications for health. Alterations in the resilience of ecosystems and shifts in patterns of disease emergence and spread manifest themselves when we fail to recognize this relationship
- To include wildlife health science as an essential component of global disease prevention, surveillance, monitoring, control, and mitigation.
- To devise adaptive, holistic, and forward-looking approaches to the prevention, surveillance, monitoring, control, and mitigation of emerging and resurging diseases that fully account for the complex interconnections among species

To achieve the goals of One Health, we will need to break down the silos between human health and veterinary medicine and to ensure effective stakeholder engagement. In other words, we need to enhance collaboration and cooperation between all parties through the development of an integrated approach to human, animal and ecosystem health. We have made some progress since the Manhattan Principles. The World Bank, the European Union, ASEAN, and other multi-national organizations have assisted in the successful development of regional One Health networks and national and regional activities. On a national level, we see an increasing number of excellent One Health activities, notably in South-East Asian countries like Thailand, Laos, Cambodia, Mongolia and Bhutan, as well as in African countries.

Connections between One Health Science and Global Health Security are being established and thus there is more attention for One Health Science with an impact on Global Health Security in governments and international institutions. That leads subsequently to increased inter-sectoral collaboration.

The One Health Platform stresses the need for basic, advanced and continued alertness and education in the area of biosecurity. International collaboration, education and coordination, using all available technology (classic and novel) are of key importance for future control of outbreaks.

In its conferences, the OHP will continue to focus on

- Highlighting contributions related to biosecurity
- Education of young scientists worldwide (fellowships for conferences)
- Special sessions and lectures about OH related biosecurity

The One Health Platform stays committed to exchanging the latest high-level scientific state-of-the-art research and gather information on OHS and AMR at the biennial congresses and disseminate the results and insights of existing and new research projects on zoonoses, emerging infectious diseases and antimicrobial resistance, including the ecological and environmental factors which drive and impact on these diseases. In that way, we aim to make science evolve, thus improving health and security. There is a clear need for the identification and prioritization of research gaps in the fields of zoonoses, emerging infectious diseases and antimicrobial resistance, including the ecological and environmental factors which impact on these diseases, and advocate the resulting scientific research agenda - both at the scientific and the policy level. We need to create synergies and facilitate the sharing of data between researchers and research groups in order to mend the prioritized research gaps and translate these novel data to anyone who might benefit from them.

The One Health Platform is committed to keep on being a strategic forum for researchers, early career investigators, governmental and non-governmental institutions, international organizations and companies in the One Health arena to foster cross-sectoral collaborations.

We stand for collaborative, multisectoral and transboundary approaches that tear down silos and enable interdisciplinary and multisectoral solutions to tackle major global health security challenges.

A One Health approach, focusing on emergent infectious diseases, which looks at health in the context of human, animal and environment relationships is the only and necessary approach in our interconnected and fast-paced world.

## Data Availability

Not applicable.

## Footnotes

1 National legislation, policy and financing; IHR coordination, communication and advocacy; Antimicrobial resistance; Zoonotic disease; Food safety; Biosafety and biosecurity; Immunization; National laboratory system; Surveillance; Reporting; Human resources; Emergency Preparedness; Emergency response operations; Linking public health and security authorities; Medical countermeasures and personnel deployment; Risk communication; Points of entry; Chemical events; and Radiation emergencies.

2 *Laureate of the 1996 Nobel Prize in Physiology or Medicine (with Rolf M. Zinkernagel), Prof. Doherty is the first veterinarian to be so recognized.*
